# On holes and strings: Earliest displays of human adornment in the Middle Palaeolithic

**DOI:** 10.1371/journal.pone.0234924

**Published:** 2020-07-08

**Authors:** Daniella E. Bar-Yosef Mayer, Iris Groman-Yaroslavski, Ofer Bar-Yosef, Israel Hershkovitz, Astrid Kampen-Hasday, Bernard Vandermeersch, Yossi Zaidner, Mina Weinstein-Evron

**Affiliations:** 1 The Steinhardt Museum of Natural History and Institute of Archaeology, Tel Aviv University, Tel Aviv, Israel; 2 Peabody Museum of Archaeology and Ethnology, Harvard University, Cambridge, Massachusetts, United States of America; 3 Zinman Institute of Archaeology, University of Haifa, Haifa, Israel; 4 Formerly of the Department of Anthropology, Harvard University, Cambridge, Massachusetts, United States of America; 5 Department of Anatomy and Anthropology, Dan David Center for Human Evolution and Biohistory Research, The Shmunis Family Anthropology Institute, Sackler Faculty of Medicine, Tel Aviv University, Tel Aviv, Israel; 6 UMR 199, PACEA, Université de Bordeaux, Pessac Cedex, France; 7 Institute of Archaeology, The Hebrew University of Jerusalem, Jerusalem, Israel; Max Planck Institute for the Science of Human History, GERMANY

## Abstract

*Glycymeris* shell beads found in Middle Palaeolithic sites are understood to be artifacts collected by modern humans for symbolic use. In Misliya Cave, Israel, dated to 240–160 ka BP, *Glycymeris* shells were found that were neither perforated nor manipulated; nevertheless, transportation to the cave is regarded as symbolic. In about 120 ka BP at Qafzeh Cave, Israel, modern humans collected naturally perforated *Glycymeris* shells also for symbolic use. Use-wear analyses backed by experiments demonstrate that the Qafzeh shells were suspended on string, thus suggesting that the collection of perforated shells was intentional. The older Misliya shells join a similar finding from South Africa, while the later-dated perforated shells from Qafzeh resemble other assemblages from North Africa and the Levant, also dated to about 120 ka BP. We conclude that between 160 ka BP and 120 ka BP there was a shift from collecting complete valves to perforated ones, which reflects both the desire and the technological ability to suspend shell beads on string to be displayed on the human body.

## Introduction

*Homo sapiens* evolved in Africa beginning at least about 200,000 years ago [1,2] and recent evidence suggests an even earlier appearance with specimens from Jebel Irhoud dated to about 300 ka BP [3]. These findings are backed by genetic evidence [4]. Early modern humans migrated out of Africa as early as 194–177 ka BP, as evident from their presence at Misliya Cave, Israel [5,6] and Apidima Cave, Greece [7]. The next physical evidence for the presence of modern humans outside of Africa is known from Skhul Cave [8–10] and Qafzeh Cave, Israel [11–13], as well as Fuyan Cave, Daoxian, China [14].

That modern humans exhibited symbolic behavior is by now well established [15–18] and the use of mollusc shell beads is an expression of this behavior is also well documented [19–21]. Shell beads from the Middle Palaeolithic or Middle Stone Age, dating to 120,000–70,000 years ago are known from three geographic regions: the Levant, North Africa and South Africa [22]. In the Levant, the shell beads found at Skhul Cave dated to between 135 and 100 ka BP, making them among the earliest ever found [23,24]. Though naturally perforated, the shells from Qafzeh Cave were suspended [25,26].

The best examples for early shell assemblages from North Africa are the caves of Contrebandiers [22] and Taforalt [27] both within Marine Isotope Stage (MIS) 5, with a date of 115±3 ka BP for the former, and 82 ka BP or earlier for the latter. Like Skhul and Qafzeh, these sites were occupied by modern humans. They were the ones responsible for the collection and use of the shells in both North Africa and South Africa, where a number of slightly later sites contained shell beads—in particular, Blombos, Sibudu and Border Caves [21, 28, 29] dating to around 80–70 ka BP.

Two other sites stand out in the list of locales containing marine mollusc shells of considerably older age: Pinnacle Point in South Africa, dated to about 160,000 years ago [30] and Misliya Cave on Mount Carmel, dated to between ca. 240,000 and 160,000 years ago [5,6,31]. At neither site were any of the symbolic shells perforated: At Pinnacle Point non-perforated *Glycymeris connollyi* shells were present and at Misliya Cave [32] *Glycymeris nummaria* (details in S1 Text in S1 File) were found.

The aims of the current research are: First, to describe the shell assemblage from Misliya Cave. Second, to apply use-wear analysis of the *Glycymeris* shells from Qafzeh Cave and Misliya Cave, coupled with a detailed experimental program to detect and catalogue microwear traces on bivalves, to test an earlier (but contested) claim that the Qafzeh shells were suspended. Third, to examine the possibility that early Middle Palaeolithic humans collected naturally perforated shells in order to display them as body ornaments, as a means of communication. This behavior was facilitated by the development of string, probably related to change in the style of clothing, apparently between 160 and 120 ka BP, thus the move from non-perforated shells to perforated ones was apparently a two-stage process.

## Materials and methods

Mollusc shells were identified to the species level based on comparative material of the mollusc collection of The Steinhardt Museum of Natural History, Tel Aviv University and the WoRMS online database [33].

The analytical protocol used in this research applies the methodological framework of use-wear analysis, comprising a unique program that was formulated to address two main issues in the study of shells: a. To formulate a data set of attributes, borrowed from protocols employed in use-wear analysis of flint [34] and ground stone [35, 36] tools in order to define wear traces on shells; b. To examine whether shells can produce diagnostic traces that may be used to make inferences about the material that had been in contact with the shells or the function of the shell.

In the first set of experiments shells were systematically abraded against various types of materials (fibers, sand, leather, reed, wood, clay, stone, etc.) to produce a catalogue of wear patterns. Wear patterns were defined by their characteristics, looking specifically at polish, striations and pitting. Polish was defined by its topography, distribution, reflectivity and texture; striations were defined by their length, width and depth; and pits were defined by their size, shape, depth and distribution. In the final stage, wear patterns were documented using light microscope cameras and a scanning electron microscope (SEM).

A second set of experiments was aimed specifically at studying traces produced through the use of perforated shells as strung items. We produced strings from wild flax, tied them through the natural holes in *Glycymeris* by various methods, and put them in simulative settings where they hung loosely or were strung with knots, to create wear patterns produced through different binding modes. The patterns created by the strings and by shells rubbing against each other were then observed and documented (details in S1 Methods in S1 File).

Shells used in the experiments were obtained from The Steinhardt Museum of Natural History, Tel Aviv University, Tel Aviv, Israel. The shells from Qafzeh Cave are on loan from the Israel Museum, Jerusalem, Israel. The shells from Misliya Cave are under study at the Zinman Institute of Archaeology, University of Haifa, Haifa, Israel. No permits were required for the described study, which complied with all relevant regulations.

## Results

### The experimental program

The two series of experiments (details in SI) produced a data set of wear patterns that clearly reflected the process to which they had been subjected. Wear patterns consist of diagnostic features that indicate the hardness, elasticity and texture of the materials in contact (fibers, sand, leather, reed, wood, clay, stone), which are clearly distinguished on a macroscopic and microscopic level. This was compared to wear patterns observed on the Qafzeh shell beads, as described below.

## Misliya Cave

### The site and its shell assemblage

**Fig 1:**
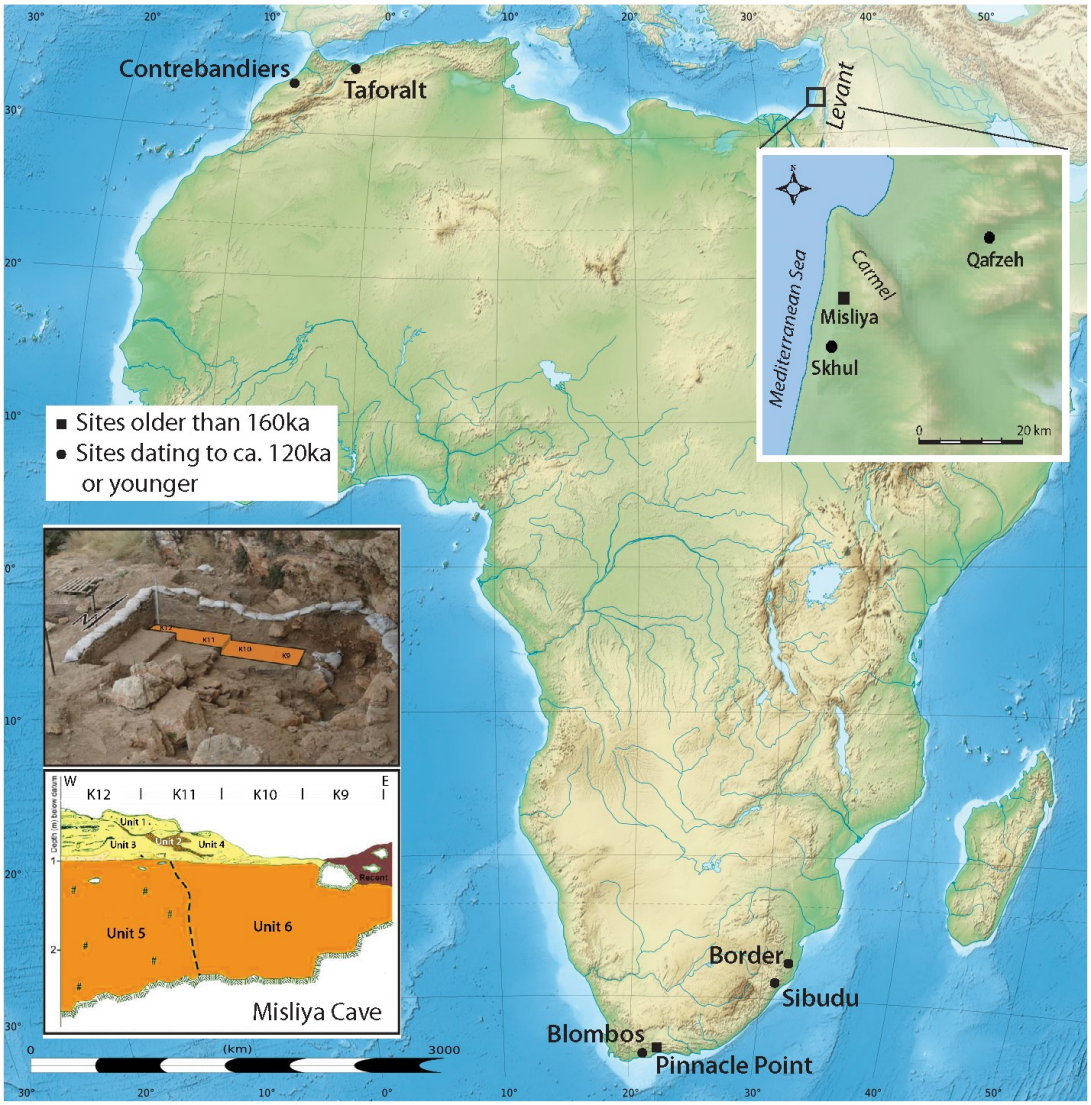
Map of sites mentioned in the text and the location of Misliya and Qafzeh caves. Bottom left: Misliya excavation area and stratigraphy (insets after Hershkovitz et al. 2018 [5]).

**Fig 2:**
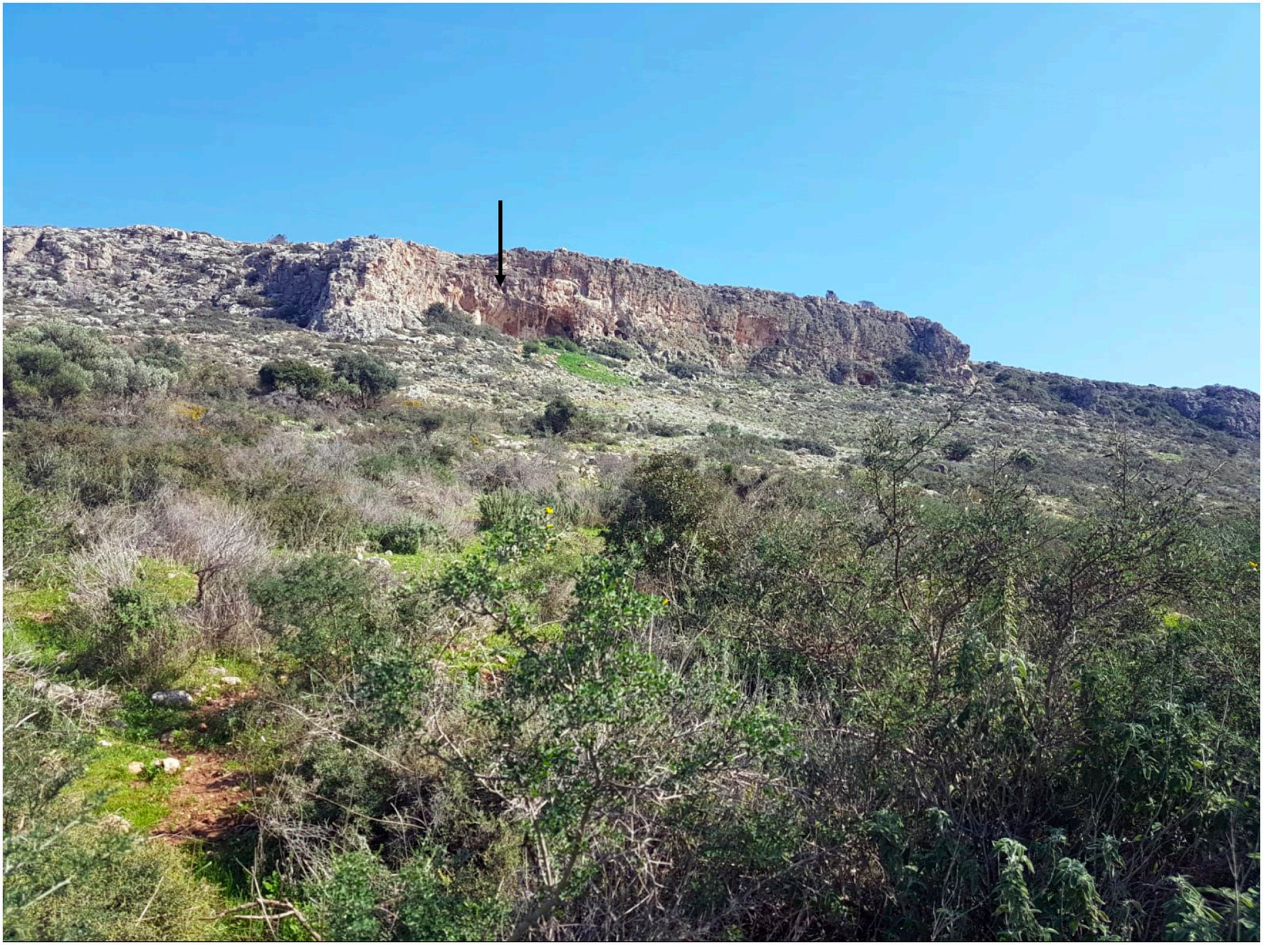
The collapsed Misliya Cave (marked by arrow) at the top of a steep slope, looking east. (Photo: Mina Weinstein-Evron).

**Fig 3:**
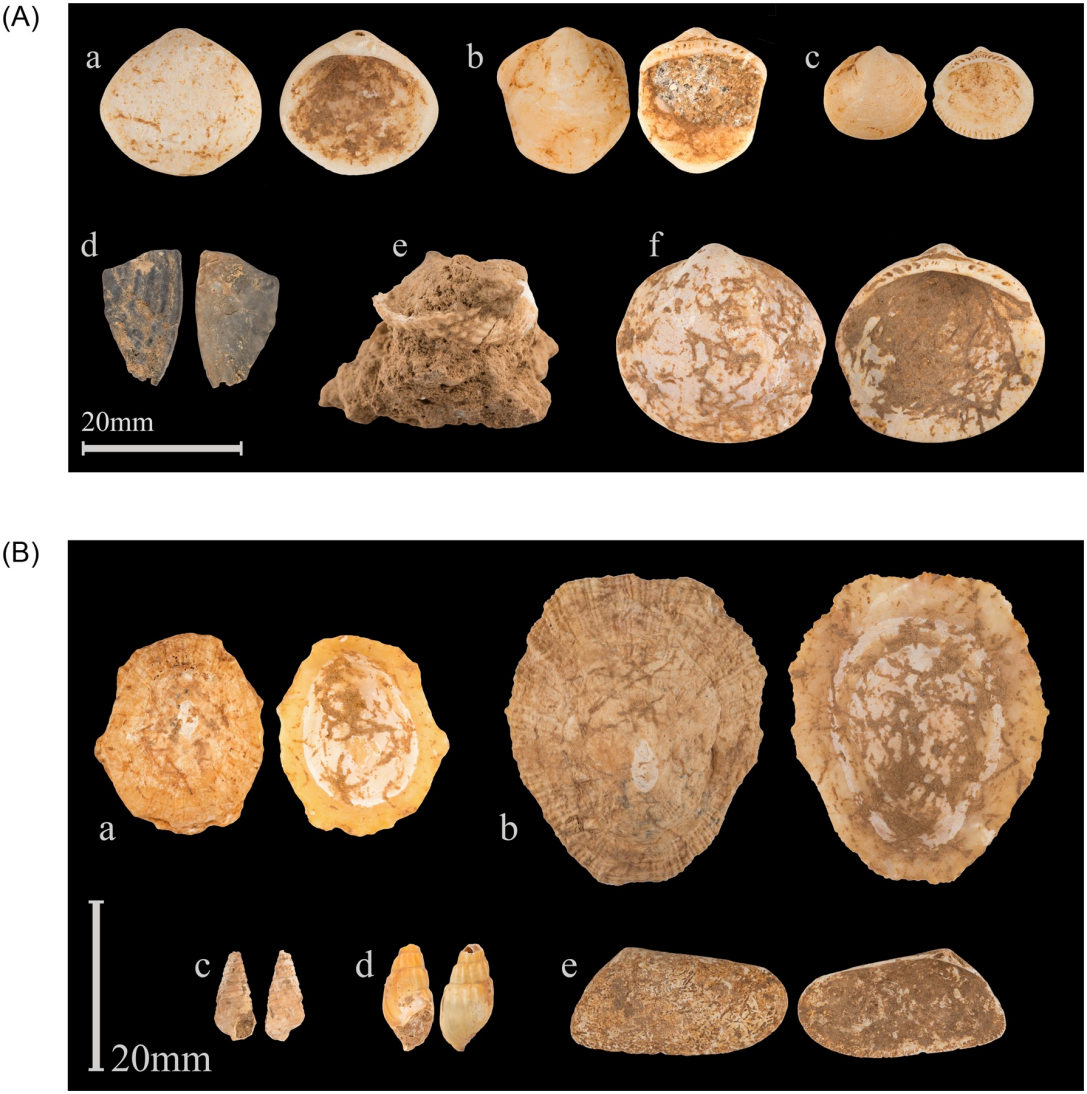
Shells from Misliya Cave. A: Shells of symbolic function: a-c, f: *Glycymeris nummaria*; d-e: *Cerastoderma glaucum*. B: Other mollusc shells that were transported into the cave: a-b: *Patella caerulea*; c: *Potamides conicus*; d: *Melanopsis lampra*; e: *Donax trunculus*. (Photos: Oz Rittner).

Misliya Cave, Mount Carmel (Fig 1), is located at the top of a steep slope 90 m above msl (70 m above its surrounding surface), its entrance facing westward, overlooking the Mediterranean Sea (Fig 2). With a “Tabun D-type” lithic industry (early Mousterian) [32] and thermoluminenscence (TL) dates of 240–160 ka BP [5,6,31] it yielded eleven human-transported shells. The molluscan assemblage (S1 Table in S1 File) consists of *Glycymeris nummaria* and *Cerastoderma glaucum* of ornamental or symbolic value. In addition, a few edible species or ecofacts were present (Fig 3; details in S1 Text and S1 Table in S1 File). The four valves of *G*. *nummaria* exhibit wear resulting from having been washed ashore as empty shells after the death of the molluscs [37]. The shells found at the cave are slightly damaged and abraded, and one was broken and then abraded, but none were perforated. One was found in the uppermost archeological layer close to the surface, another was discovered in Unit 4 and two more came from Unit 6 of the excavation, both units attributed to the Early Middle Palaeolithic (EMP) based on both lithics and absolute dates (See S1 Text in S1 File). A burnt fragment of a *C*. *glaucum* shell was found in Unit 6 as well, while another was embedded in breccia and only a part of the valve could be seen (Fig 3a and 3e), but the brecciated context confirms its association with EMP levels.

### Microwear analysis

The Misliya Cave shells exhibit no traces that reflect human manipulation. Neither polish nor striations are visible, except for an isolated patch of polish on top of the umbo of one of the valves. This evidence is consistent with a taphonomic process in bivalves whereby valves rub against each other during the lifespan of the mollusc.

## Qafzeh Cave

### The shell assemblage

**Fig 4:**
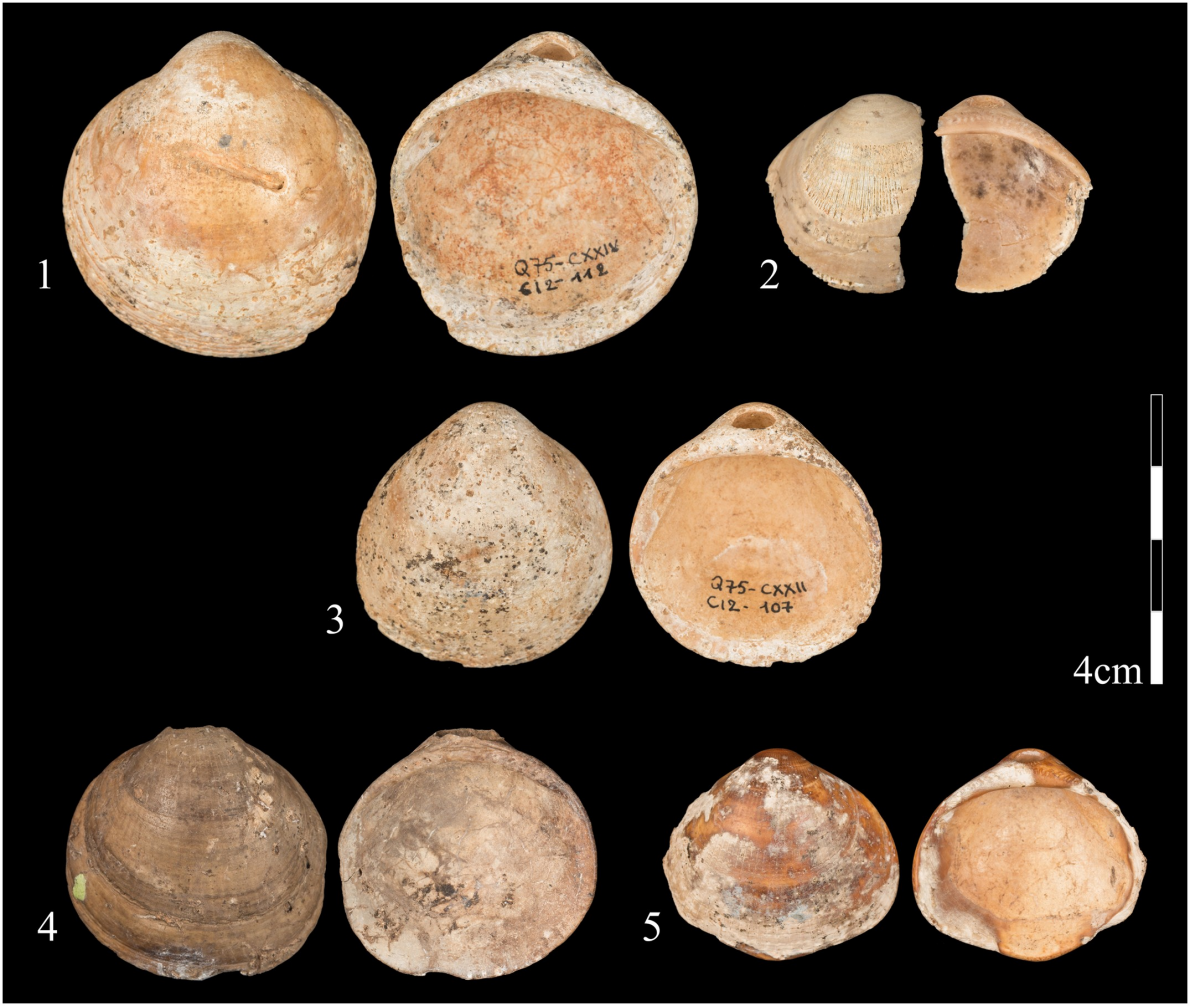
Shells from Qafzeh Cave on which use-wear was studied. 1. specimen 112; 2. specimen 102; 3. specimen 107; 4. specimen 404; 5. specimen 632. (Photos: Oz Rittner).

Ten *Glycymeris* shells were discovered at Qafzeh Cave, in Layers XXI-XXIV, immediately under human graves. They include seven complete or almost complete valves with perforations in the umbo, and a few fragments, all of which have been described in detail previously (Fig 4) [26].

### Microwear analysis

Five shells were selected for microscopic examination due to their good state of preservation. These shells showed traces that were produced by contact with a string, coloring treatment with ochre and traces of shell-to-shell contact, all of which indicate that the valves had been arranged on a string (Table 1).

**Table 1:** Summary of the results of the use-wear analysis of the Qafzeh shells.

No. Fig 3a	SPECIMEN*	WEAR CONSISTENT WITH FIBRE SUSPENSION	COLORING TRACES	CONTACT WITH ANOTHER SHELL
1	112 [1, XXIV]	√	√ (with residue)	√ at interior margin
2	102 [7, XXIV]	√	none	none
3	107 [2, XXII]	√	√ (with residue)	√ at interior margin
4	404 [5, XXI]	√	√ (without residue)	√ at interior and exterior margin
5	632 [6, XXI]	√	√ (without residue)	√ at interior face near the umbo

*Specimen is the catalogue no. given by the excavators, followed by their number in Bar-Yosef Mayer et al. 2009 [26]: Pl. 1, and Roman numerals refer to layer in the excavation.

**Fig 5:**
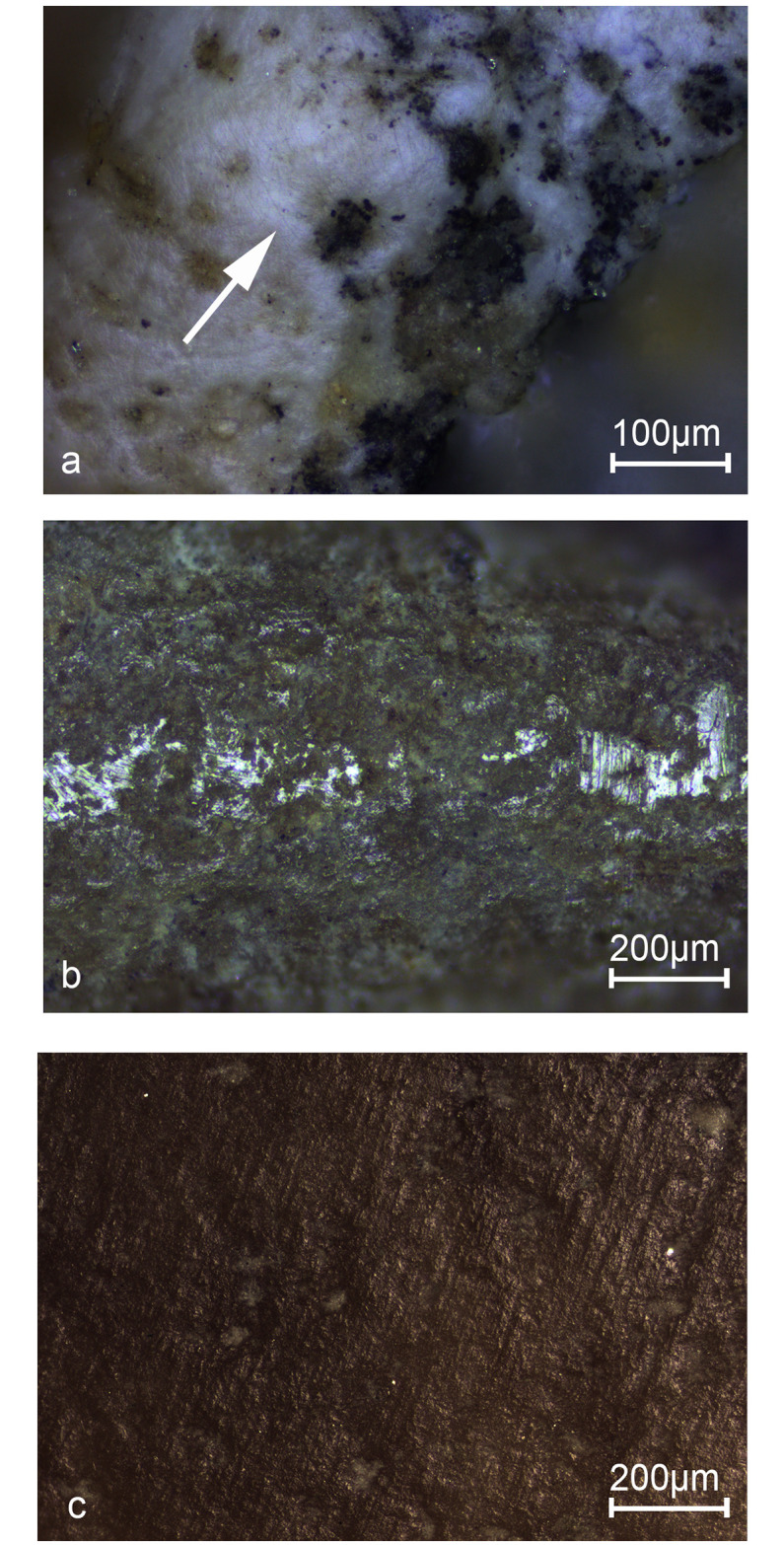
Use wear on shells from Qafzeh. a: fine striations near the hole between the umbo and the center of the hinge. b: Polish patches produced by valve-to-valve contact. c: Traces associated with colorant. (Photos: Use-wear Laboratory, Zinman Institute of Archaeology, Iris Groman-Yaroslavski).

All shells exhibited fine striations (visible at magnifications of 100-200x) near the hole between the umbo and the center of the hinge (Fig 5a; S1b Fig in S1 File). The orientation of the striations was parallel to the long axis of the shell, corresponding to the pattern observed on the experimental shell.

Polish patches produced by valve-to-valve contact were observed on four of the five shells (Table 1; Fig 5b). These isolated patches were distributed along the margins of the shells. The tiny striations appearing on their surfaces were probably produced by grains originating from a red substance, previously identified as ochre [38], which was used to color the shells (clearly visible on two of them, nos. 112 and 107). Based on the distribution pattern of the patches we were able to reconstruct the arrangement of the valves that had rubbed against each other concave side to convex side. A similar rubbing pattern was produced in our experiment. Considering that no such traces were observed on the center of the concave face of the shells, it is reasonable to assume that the shells had touched each other only at their margins.

Traces associated with colorant were observed on four shells (Fig 5c): A wide surface covered with striations indicates that the shell was rubbed against the colorant. The fifth shell had no residue of colorant.

To conclude, our analysis shows that the Misliya shells bore only use-wear consistent with natural abrasion, while the Qafzeh specimens manifested evidence of stringing near the holes, the use of ochre and contact between shells that were strung adjacent to each other.

## Discussion

The presence of bivalve shells at sites containing early modern human remains comes as no surprise, as they are considered a hallmark of modern human behavior [17, 39]. Their social role is significant, possibly marking the wearer’s place in kinship networks, marital status and group affiliation. They may have served as a type of charm with apotropaic meanings. The choice of a smooth, round object as a social signal is probably not accidental, because primates’ brains respond favorably to curved forms, including concentric circles [40, 41]. Nonetheless, in order to function as a means of interpersonal communication and provide information about individual or group identity, the shells must not only have a symbolic meaning understood among different groups, they must also be displayed in a way that is clearly visible to others.

Two shell assemblages were chosen to study the notion of shell bead display by humans and its timing: Misliya Cave, dated to ca. 240–160 ka BP [5,6] and Qafzeh Cave, dated to 92±5 ka BP [42] or earlier, ca. 120 ka BP, based on other studies that correlate Qafzeh with MIS 5e [43, 44 and see 45, 46]. In both sites, empty and naturally abraded valves of *Glycymeris nummaria* had been collected from the Mediterranean shore by modern humans. The Misliya shells were not perforated. The Qafzeh shells included seven with the umbo intact, and all had naturally perforated holes ranging from ca. 3–6 mm in diameter [26].

The Misliya shells show no evidence of human manipulation, apart from having been collected and transported to the cave. The *Glycymeris* shells from Qafzeh Cave were naturally perforated, and had notches that were assumed to have resulted from their suspension [26]; however, a recent study demonstrated that these notches are part of the variability of naturally abraded holes of *Glycymeris*, i.e., not indicative of threading [47]. But in our current use-wear experimental analysis (see S1 Text in S1 File) testing various materials rubbing against such shells, the microwear pattern of strung shells corresponds to those observed on five of the best preserved Qafzeh shells, supporting our previous notion that the shells had been suspended.

Shells discovered to date in the Middle Palaeolithic/Middle Stone Age cave sites of Skhul, Qafzeh, Blombos, Border, Sibudu, Taforalt, Contrebandiers and others are dated to between 120 and 70 ka BP, and most are perforated. Ancient collectors relied mainly on naturally perforated and abraded shells and it would be reasonable to assume that much like the Qafzeh shells, those were also strung in order to be displayed [21]. Nonetheless, in two Middle Palaeolithic (or Middle Stone Age) sites, Pinnacle Point and Misliya Cave, dated to 164 ka BP (±12 ka) and 240–160 ka BP, respectively [5, 6, 30, 31] the shells were not perforated. Curiously, in both cases the shells that were collected belong to the genus *Glycymeris*, and in both sites the shells were rather small and abraded [31].

The desire of some of the earliest modern humans to collect shells may have triggered the search for a way to display them to others, and the already-invented string enabled such exhibition. Physical remains of fibers have been discovered at Dzudzuana (dated to ca. 30 ka BP) [48], where they were spun and dyed, and at Abri du Maras (dated to ca. 80 ka BP [49], where fiber fragments were twisted. At the latter site, a cord fragment adhering to a flake was recently discovered and dated to about 46–40 ka BP [50]. Circumstantial evidence for string use comes from Regourdou, where wear on a tooth suggests the manipulation of cord ca. 72 ka BP [51]. Bone needles, related to the use of string and considered to indicate the use of clothing, first appeared in about 45 ka BP, yet additional indications for the use of clothing date to at least 80 ka BP [52]. These examples are all dated to later than 100 ka BP; and additional evidence for the appearance of strings, cords or fibers indicates even younger occurrences [53, 54]. There are other claims for strung and perforated ornaments earlier than 100 ka BP [55, 56] but the chrono-stratigraphic context of the examples they provide have not been confirmed [57]. The only persuasive case is the recent reexamination of eagle talons from Krapina that revealed a fiber fragment attached to a talon, apparently dating to 130 ka BP [58]. To date, this find has no parallel. This discovery reinforces our notion of a connection between the emergence of string and the emergence of perforated shell beads.

The reasons for shell collection in the EMP could have varied from deep symbols relevant to the protection of life [59] to identity representation. Specific focus on bivalves (*Glycymeris* and *Cerastoderma* in the case of Misliya) may have been due to mental templates inherent in all humans. They may represent cosmic powers, but also the mere notion of life itself as originating in the sea. Smooth and round bivalves with their subconscious connotations have a universal appeal.

While these suggestions are based on psychoanalytical research [59], an alternative explanation could be related to the abundance of the species (the Levantine coast’s malacofauna composition in the MIS5e is not known to us) or simply to the attraction of the color or pattern on the natural shells. From as early as the Lower Palaeolithic [60] humans collected shells and carried them to habitation sites, yet around 120 ka BP, they started collecting perforated shells. Because in current thanatocoenosis about 40% of *Glycymeris* shells are naturally perforated [37], the fact that almost all of the specimens found in the archaeological sites are perforated, albeit naturally, suggests their collection is intentional and is meant to enable their stringing and display. On the other hand, the fact that all shells from two earlier Middle Palaeolithic assemblages are not perforated, suggests that there was neither need, nor intention or possibility of their display, and they were collected for their “face value”, but without necessarily showing them to others.

The ability to display shell beads depended on their suspension on string, directly on the human body or by attachment (sewing) shells onto clothes. We thus come a step closer to finding out the date when the use of string was first introduced. Our data suggest that sometime within the time range of 160 and 120 ka BP the technology for making strings emerged, and that this technology boosted the collection of naturally perforated shells for display, a practice common to this day.

## Supporting information

S1 File (DOCX)
