# Supplementary material for: On holes and strings: Earliest displays of human adornment in the Middle Palaeolithic
Source: PLoS One. 2020 Jul 8;15(7):e0234924. doi: 10.1371/journal.pone.0234924 (PMC7343129; doi:10.1371/journal.pone.0234924)
Supplement: S1 File — (DOCX) [file pone.0234924.s001.docx]

**S1 text: SUPPORTING INFORMATION**

**ON HOLES AND STRINGS: EARLIEST DISPLAYS OF HUMAN ADORNMENT**

**IN THE MIDDLE PALAEOLITHIC**

Daniella E. Bar-Yosef Mayer, Iris Groman-Yaroslavski, Ofer Bar-Yosef, Israel Hershkovitz, Astrid Kampen-Hasday, Bernard Vandermeersch, Yossi Zaidner, Mina Weinstein-Evron

**I. Misliya Cave: A brief description of the site and its main finds**

Misliya Cave is located on the western slopes of Mount Carmel overlooking the Mediterranean Sea, south of Nahal (Wadi) Sefunim at an elevation of ca. 90 m on the upper part of a steep slope. The site is situated 12 km south of Haifa (Fig 1), and 7 km north of Nahal Me‘arot (Wadi el-Mughara) with the famous caves of Tabun, Skhul, el-Wad and Jamal [8, 9, 46, 61, 62].

Misliya Cave contained layers rich in finds from the late Lower Paleolithic (Acheulo-Yabrudian) and Early Middle Paleolithic (EMP, Early Levantine Mousterian) [32, 63]. The site is a large collapsed cave which today appears as a rock shelter (Fig 2). Strongly cemented archaeological sediments (breccia) are found on three terrace-like surfaces at the base of the cliff, all sloping gently to the west (henceforth the Upper, Middle and Lower Terraces). The Lower Terrace yielded only Acheulo-Yabrudian finds *in situ* [32, 63] whereas on the Upper Terrace, EMP remains were found. In the northeastern part of the Upper Terrace, brecciated layers change laterally into softer sediments. This area (Fig 1), where a series of well-preserved MP hearths and abundant lithics and animal bones were unearthed, was the main focus of the excavation and yielded the shells reported here.

The archaeological sequence of the Upper Terrace, is 1-1.5 m deep, and was divided into six stratigraphic units. Units 1 and 2 represent surface eroded breccia and a later terra rossa intrusion, respectively. Units 3 and 4 represent well-preserved but residual MP habitation layers rich in lithics and faunal remains and containing combustion features. The most extensive units are Units 5 (brecciated) and 6 (the soft sediment area). The lithic analysis of the MP assemblages from all stratigraphic units of Misliya Cave [32, 63, 64] shows that they all belong to the Early Levantine MP (“Tabun D-type” industry) [8, 65, 66]. The EMP layers of the site were dated to ca. 240-160 ka BP (with one outlying date of 140 ka BP) [5, 6, 13]. These dates correspond to MIS 7-6, when the coastline was located 2 km west of its current location and are roughly within the time range of the same cultural phase at the nearby Tabun Cave [67] and at Hayonim Cave, in the western Galilee [68, 69]. A broken maxilla, found in Unit 6, represents one of the earliest known modern humans outside Africa, was confined to an age range of 194-177 ka BP [5, 6]. The shells, mostly derived from Units 4 and 6 (Table SI:1) represent the wider chronological framework of the site.

**II. Detailed description of the molluscan assemblage of Misliya**

The Misliya shells are described and interpreted below to complement S1 Table and Fig 3.

**S1 Table**: The molluscan fauna of Misliya in taxonomic order

| **Species** | **height** | **width** | **Observations** | **Stratigraphic position** | **Geographic origin** | **Habitat of the species** |
| --- | --- | --- | --- | --- | --- | --- |
| *Patella caerulea*  Linnaeus, 1758 | 38.27 | 31.86 | Small damage at margin | Unit 6 | Mediterranean | Tidal rocks |
| *Patella caerulea* Linnaeus, 1758 | 23.65 | 19.88 | Slight damage at margin | Unit 6 | Mediterranean | Tidal rocks |
| *Melanopsis lampra* Bourguignat, 1884 |  |  | Top whorls broken | Unit 4/5 | Local freshwater | Coastal rivers |
| *Potamides conicus* (Lozouet, 1986) | 10.08 | 4.33 | Missing first whorl | Unit 6 | Mediterranean | Estuaries and lagoons |
| *Glycymeris nummaria* (Linnaeus, 1758) | 25.49 | 26.58 | Slight damage at margin, slight abrasion to teeth at margin and to hinge teeth | Surface | Mediterranean | Sea bottom, washed ashore |
| *Glycymeris nummaria* (Linnaeus, 1758) | 15.84 | 17.24 | Tiny hole in umbo, too small for stringing , all margins abraded, relatively young specimen | Unit 4 | Mediterranean | Sea bottom, washed ashore |
| *Glycymeris nummaria* (Linnaeus, 1758) | 12.18 | 13.29 | Slight damage at margin, all teeth fresh as well as exterior concentric lines | Unit 6 | Mediterranean | Sea bottom, washed ashore |
| *Glycymeris nummaria* (Linnaeus, 1758) |  |  | Broken valve, and abraded, hinge and umbo intact | Unit 6 | Mediterranean | Sea bottom, washed ashore |
| *Cerastoderma glaucum* Bruguière, 1789 | 16.02 | 14.99 | Embedded in breccia | Not available | Mediterranean | Brackish water |
| *Cerastoderma glaucum* Bruguière, 1789 |  |  | Burned fragment, 14.73 mm long | Unit 6 | Mediterranean | Brackish water |
| *Donax trunculus* Linnaeus, 1758 | 13.35 | 27.53 |  | Unit 2 | Mediterranean | Sand; tidal & subtidal |

*Patella* *caerulea* (Fig 3b:a, b): Two specimens were discovered, one fairly large, measuring 31x38 mm, the other smaller, measuring ca. 23x19 mm. Both specimens are almost complete, but each of them is slightly damaged at the edge of the margin. This damage may have resulted from forceful removal of the animal from the tidal rock to which it was attached. The notion of forceful removal of *Patella* specimens has been proposed, but is debated and requires further experimentation [70, 71]. *Patella* *caerulea* is an edible species but the only other occurrence of *Patella* in an MP site in the Levant is a fragment from Ras el Kelb [72]. This species is also encountered in shell middens at Middle and Upper Palaeolithic (UP) sites throughout the circum-Mediterranean [73, 74]. The occurrence of *Patella* shells in small quantities at sites that are walking distance from the coast, but not right on it, suggests the possible presence during the site’s occupation of a shell midden on the seashore, which is probably now submerged [75]. The few specimens brought to the top of the escarpment where Misliya is located may have been taken there as an “extra snack”, but they must have been consumed shortly after collection to avoid food poisoning. Empty shells of previously consumed shellfish may have also been brought to the site as a “souvenir”, or to show what food was obtained on the coast.

*Melanopsis* *lampra*: This is a freshwater snail inhabiting the coastal streams of Israel [76]. While this shell might have been accidentally brought to the site by water fowl [77], it may also have been inadvertently brought by humans. It is known from other archaeological sites to which it was presumably taken along with drinking water [23]. This suggests that already in the early phase of the MP, containers were used to bring water to the site, which meant that humans did not necessarily have to walk to a water source for drinking. This in itself may be yet another trait of human behavior not previously observed. Ostrich egg shells, the fragments of which were discovered at the site [78] may have served as water containers, as has been suggested for later sites in South Africa [79]. Containers made of hide or wood may also serve this purpose; the processing of both materials was identified on the lithics from the site [80].

*Potamides conicus*: This snail typically resides in brackish water and is often found in estuaries or lagoons. In North Africa it is also known to live in inland waters of saline and hypersaline environments. *P. conicus* was collected in brackish water, but was neither perforated and intended for use as ornament, nor consumed. In the case of Misliya, there are two possibilities for its having entered the cave: by means of birds or other animals, as explained above for *M. lampra* [77], or by humans. If humans were responsible, the presence of this species, along with *C. glaucum* that lives in the same environment and was also found at Misliya (see below), would shed light on the possible value of exploring brackish water environments, which are often ignored in the archaeological record. Brackish water environments can be the source for sedges and other aquatic and hydrophilous plants that may have served as multiple resources for producing various everyday artifacts and could have served as raw material for making baskets or nets. Salt is another resource that could have been procured in such an environment.

*Glycymeris nummaria* (previously called *G. insubrica*, or *G. violacescens*): Four valves of this species were found. They are naturally abraded, which shows that all were collected from the beach rather than from the sea. One specimen that seemed less abraded is a juvenile specimen; another was broken prior to its abrasion; another had a tiny hole (diameter 0.46 mm) in its umbo that is a result of abrasion, but the hole is too tiny to have served for stringing. *G. nummaria* lives in considerable depths in the ocean (ca. 30 m), but is commonly found washed onto the shore [34]. One of the *Glycymeris* shells (Fig 3a:f) comes from the surface of the site but the others are from secure EMP contexts.

*Cerastoderma glaucum*: This bivalve, represented by two specimens, inhabits brackish waters along the Israeli coast. It is known from various estuarine and lagoon environments [81, 82] and coexists with *P. conicus* (discussed above). The complete *C. glaucum* unfortunately lost the correct label for matching with the stratigraphy, but because it is embedded within a lump of brecciated sediment (Fig 3a:e), we are confident of its association with the EMP as well. This shell, like the *Glycymeris* specimens, may have had symbolic value. Other early occurrences of shells from the Cardiidae family are known, for example, from Skhul Cave [7: p. 224] and el Harhoura in North Africa [22].

*Donax trunculus*: This Mediterranean bivalve lives buried in sand in the tidal and subtidal zone [83]. Because *Donax* sp. is encountered in Middle Stone Age contexts in South Africa, unmistakably associated with modern humans [31], its presence at Misliya is no surprise, but the single shell from Unit 2 might also represent later human activity.

**III. The experimental program for *Glycymeris* shell use-wear**

**III.1. Introduction**

In order to interpret use-wear patterns of *Glycymeris* shells that were collected by the MP human populations of Misliya and Qafzeh caves, presumably as ornaments, it was necessary to conduct experiments with similar shells. These would validate the microscopic traces observed on the archaeological specimens.

The microscopic analysis and experimentation were conducted at the Use-wear Analysis Laboratory of the Zinman Institute of Archaeology, University of Haifa. The analysis protocol used in this research was formulated with the aim of addressing two main issues in the study of shells:

a. To formulate a set of attributes, borrowed from protocols employed in use-wear analysis of flint [35] and ground stone [36, 37] assemblages, encompassing the macro and micro characteristics of the traces, to define wear traces on shells.

b. To examine whether shells produce diagnostic traces that may be used to infer the identity of the material that was in contact with the shells, or the function of the shell. In the first set of the controlled experiments shells were systematically abraded against various types of materials. A second set of experiments was aimed specifically at studying traces produced through wear on perforated shells that were used as strung items.

**III.2. Methods**

The analysis procedure in this research began with observations on the macro scale which were done using a Nikon SMZ 745T stereoscope at magnifications of 6.7–50x. At this stage wear features such as rounding, pitting, abrasion and fragmentation were characterized. Next, observations on the micro scale were done using a Leica DM 1750 metallurgical microscope at magnifications of 100–500x to identify polish and linear features (striations and grooves). SEM inspections complemented the above-mentioned observations to more precisely define the characteristics of the traces and determine the contribution of SEM observations to our analyses and interpretations. The conditions selected were low vacuum, at 15 KV, in back-scattering mode (BSE). Shells were placed in the chamber on a stub in the same position, with the manipulated surface horizontal to the detector. The shells were then fastened for stabilization.

S2 Table provides the list of attributes used to describe the wear traces. This list is based on Adams et al. [36] and Van Gijn [35] and includes traces that are relevant to shell material and bidirectional motion. We used *Glycymeris nummaria* shells from the Israeli Mediterranean coast from the collections of The Steinhardt Museum of Natural History. Each shell was numbered and observed with the naked eye, then examined under the microscope. Documentation of the traces was done using the microscope cameras and in some cases Helicon Focus© software was used to create full depth micrographs by Z-stacking.

**S2 Table**: List of attributes used for describing the wear patterns on the shells (Based on [35-37]).

| Topography | Flat |  |
| --- | --- | --- |
|  | Sinuous |  |
|  | Uneven |  |
| Microtopography | Flat | Regular or irregular |
|  | Sinuous | Regular or irregular |
|  | Uneven | Regular or irregular |
| Linear features | Distribution | Loose, covered, concentrated |
|  | Density | Separated, closed, connected |
|  | Incidence | On highs, on interstices |
|  | Width | Wide, narrow |
|  | Length | Long, short |
|  | Depth | Shallow, deep |
|  | Longitudinal morphology | Continuous, intermittent |
|  | Transverse morphology | V-shaped, U-shaped, flat bottom |
| Pits | Distribution | Loose scattering, closed, overlapping |
|  | Size | Tiny, small, large |
|  | Depth | Shallow, deep |
|  | Shape | Irregular, circular |
|  | Shape of margins | Rounded, sharp, irregular |
| Polish | Microtopography | Flat, sinuous, irregular |
|  | Distribution | Spread, patches, linear, reticular, capillar |
|  | Linkage | Spread, linked, non-linked |
|  | Reflectivity | Dull, bright |
|  | Incidence | On highs, on interstices |
|  | Texture | Smooth, rough |
|  | Opacity | Transparent, opaque |

**III.2.a. Experiment design: Wear attributes on shells**

The first controlled systematic experiment was aimed at creating wear features through contact with various types of material in order to examine whether shells produce diagnostic traces that may be used to infer which materials had been in contact with the shells (S1 Fig). Thirteen shells were used, each of them rubbed on a specific type of surface (S3 Table) for 15 minutes, a duration considered long enough to generate sufficient wear traces for analysis. Shells were abraded in a bi-directional motion, on their exterior face, from the highest "peak", about 1 cm from the umbo, to the margin of the valve. We selected this method so as to create broad abraded surfaces that are easier to observe and would contain the overall wear features, as opposed to using only the margins of the shells where the abraded surface is harder to observe.

**S3 Table**: List of experimental shells and the surfaces on which they were abraded, by hardness

| **Experimental shell number** | **Hardness** | **Contact materials** |
| --- | --- | --- |
| 220 | Soft materials | Flax fibers |
| 545 |  | Loose sand |
| 577 |  | Loose sand with water |
| 221 |  | Oiled leather |
| 222 |  | Leather |
| 217 | Medium hardness materials | Fresh paper reed (*Cyperus papyrus*) |
| 223 |  | Dry wood log |
| 239 |  | Almost leather-dry clay |
| 240 |  | Leather-dry clay |
| 614 | Hard materials | Sand on limestone |
| 219 |  | Limestone |
| 218 |  | Basalt |
| 246 |  | Flint |

The surface intended for manipulation was marked by a circle using a pen. Then, during observation we selected the most indicative area and marked a tiny dot near it so the surface selected would be easy to find when later observing it under SEM. Thus, at the end of the documentation process, we had three types of photos: a macrograph, a micrograph and an SEM scan, all showing the same spot.

All shells were handheld, with the right index finger on the interior face of the valve, and the material being tested lying on a flat surface. The one exception to this was the experiment using flax threads (shell #220), which was done with the shell stabilized on a flat surface and the flax string abrading the shell held by hand (S1 Fig: b).

The contact materials used were divided into three main groups according to their hardness and relative to the shell's hardness (S3 Table). The first was a group of soft materials including flax fibers, leather, oil-soaked leather and papyrus sedge. In this group we introduced another experiment: abrading the shell on loose sand in a container (one dry and one with water) to replicate wear patterns associated with a beach environment. We consider this setting to represent soft material (although some beach sand contains hard particles of quartz) because in this case the sand acts as a soft matrix as the shell sinks into it during the application of pressure. In contrast, sand on a slab of limestone was included in the group of hard materials because in this setting the limestone holds the sand particles and high pressure conditions are formed, enabling intense abrasion. The second group consisted of materials of medium hardness including almost leather-dry clay and wood, and the third group included hard materials, comprising various types of rocks: limestone, basalt and flint. These materials were chosen because they represent materials that might have been in contact with shells under varying conditions.

**III.2.b. Experiment design: ornamental binding**

The next set of experiments was aimed at creating wear patterns on shells worn as adornments on the human body (S2 Fig). To create such traces, we set up a simulation that would accelerate the wear. Ten shells with naturally abraded holes, were strung on a flax string that was produced out of natural fibers from year-old, fully dried stalks of flax (*Linum usitatissimum*) collected in the vicinity of Yavniel in the Galilee. Shells were hung loosely or strung with knots, to evaluate the wear on the hole of the shell and wear patterns created through different binding modes. The shells were strung in various configurations: One shell hung loosely on a string (S2 Fig: a); several single shells were tied with a knot around each shell (S2 Fig: b); two shells were strung together, interior-to-exterior faces (S2 Fig: c); and some shells were tied, interior-to-interior faces (S2 Fig: d).

In order to simulate human wear with the intervention of sweat from the body, the flax string was wetted every eight hours with “artificial perspiration”, using a Hartmann's solution, which is used in medical practice to replace body fluids and mineral salts (contains sodium, chloride, lactate, potassium and calcium). The string with the shells was hung in front of a fan for ten days emulating the motion of wearing the shells as a necklace (S2 Fig: e).

**III.3. Results of the controlled experiment on varying materials**

The original surface of the shells that were collected on the beach is pitted and irregular, with isolated linear features and polished surfaces that developed on protruding surfaces, distributed evenly on the entire surface (S3 Fig). No patterns that may be confused with use-wear were observed, and the comparison to the worked shells clearly showed that use-wear is unique in every aspect. The controlled experiment resulted in indicative wear patterns that could be distinguished from each other (S4-S6 Figs). In general, the wear patterns exhibited traces that are indicative of the hardness (as in the case of leather compared to limestone), texture (as in the case of limestone compared to basalt) and elasticity (as in the case of wood compared to flint), and may provide evidence of lubrication (as in the case of fresh papyrus sedge, loose sand and water and almost leather-dry clay). Most indicative of all is the polish, whose characteristics such as microtopography, reflectivity and texture provide the best evidence for the material in contact. In cases where shells were abraded in a bidirectional motion, striations are also indicative (see below). In our experiments, all the shells exhibit longitudinal, parallel-oblique striations, which are the result of this type of motion. Below we shall discuss the various features seen on the shells according to the main characteristics of the use-wear.

**III.3.a.** **Topography and microtopography**

The topography of the use-wear, studied on the macro scale reflects the hardness and elasticity of the material in contact and is visible under low magnifications (up to 50x). Observing the microtopography under high magnification (100-200x) enables additional details to be seen (S4 Table).

**S4 Table**: Characteristics of the topography and microtopography observed for the shells used in the controlled experiment.

|  | **Material in contact** | **Topography** | **Microtopography** |
| --- | --- | --- | --- |
| 220 | Flax fibers | Sinuous and uneven | Regular |
| 545 | Loose sand | Uneven | Irregular |
| 577 | Loose sand with water | Sinuous | Irregular |
| 221 | Oiled leather | Uneven | Regular |
| 222 | Leather | Sinuous | Irregular |
| 217 | Fresh papyrus | Sinuous | Regular |
| 223 | Dry wood log | Flat - sinuous | Regular |
| 239 | Almost leather-dry clay | Sinuous | Regular |
| 240 | Leather-dry clay | Flat | Regular |
| 614 | Sand on limestone | Irregular | Irregular |
| 219 | Limestone | Flat | Irregular |
| 218 | Basalt | Flat | Irregular |
| 246 | Flint | Uneven | Irregular |

Materials that are soft and elastic (S4 Fig) usually generated a sinuous topography where protruding surfaces are rounded and microtopography is regular and smooth, the result of a moderate frictional process. The softest materials, as in the case of the flax thread (S4 Fig: a:1-2), affected the high areas of the shell and to a greater extent the low areas. Exceptional was the case of loose sand, where the original topography was still evident but a granular surface was formed due to high abrasiveness of the tiny particles on both high and low areas (S4 Fig: b1-2). Compared to the wear by loose sand and water, rounding of the high areas was extremely developed, reflecting the role of water as a lubricant that slows down aggressive abrasion (S4 Fig: c1-2). The rounding enhanced by the water is somewhat similar to the rounding produced by the contact with leather (S4 Fig:c1-2), but in this case it is developed to a higher degree, reflecting both the elasticity of the leather but also its greater abrasive capacity (S4 Fig:d1-2, see also S7 Fig regarding hide compared to leather: Hide refers to a "fresh" oily substance, whereas leather is drier and less fatty).

Materials of medium hardness affected the surface of the shells more aggressively (S5 Fig). The high areas were extremely reduced, creating wide, flatter surfaces with rounded edges. Hard materials on the other hand, flattened the surface of the shell (S6 Fig), where the hardest ones (such as flint) rapidly truncated the highs resulting in an irregular topography composed of beveled surfaces and irregularly shaped pits (S6 Fig: d1-2). SEM observations provided an additional view of the topography; however, in most cases the surface seemed flatter than through a reflected-light microscope, especially where extreme rounding developed as in the case of loose sand with water (S4 Fig: c1-2) and leather (S4 Fig: d1-2). Through SEM observation, rounding was more evident at the edges of the polished surfaces or around the margins of pits; however, relying exclusively on SEM observation impedes the evaluation of the degree of wear.

**III.3.b.** **Linear features**

Striations, grooves and streaks of polish are considered linear features, observed under high magnification. Linear features mainly indicate the directionality of the action (which is constant in the case of the controlled experiment), formed by the dragging of particles loosened from the shell as well as from the material in contact with it, or scratching by the more durable protruding surfaces of the material in contact. Therefore, the characteristics of the linear features reflect the type of loosened grains, topography and hardness of the material in contact (S5 Table). These nuances are evident when comparing materials of similar hardness, as in the case of the oiled leather, which did not produce striations, compared to dry leather that created a heavily striated surface (S7 Fig). Surprisingly, this was also the case for loose sand compared to loose sand with water, probably because the sand was extremely loose, and there was insufficient pressure to scratch the shell. Materials of medium-range hardness, such as papyrus sedge, wood and clay, produced heavily striated surfaces, usually on highs and V-shaped striations. Harder rocks, such as limestone, basalt and flint altered the entire surface and formed heavily striated, flat surfaces. Flat-bottomed and U-shaped striations were occasionally created by the softer limestone and clay. SEM observations contributes greatly in this case to a better definition of the morphology of the linear features, as images at high magnification are in full focus without shadows.

**S5 Table**: Characteristics of the linear features observed for the shells used in the controlled experiment.

|  | **Material in contact** | **Distribution** | **Density** | **Incidence** | **Width - depth** | **Longitudinal morphology** | **Transversal morphology** |
| --- | --- | --- | --- | --- | --- | --- | --- |
| 220 | Flax fibers | Loose | Separated | On highs | Narrow-shallow | Continuous | V-shaped |
| 545 | Loose sand | None | None | None | None | None | None |
| 577 | Loose sand with water | None | None | None | None | None | None |
| 221 | Oiled leather | None | None | None | None | None | None |
| 222 | Leather | Covered | Closed | On highs | Wide-deep | Continuous | V-shaped |
| 217 | Fresh papyrus | Covered | Closed | On highs | Narrow-deep | Continuous | V-shaped |
| 223 | Dry wood log | Covered | Closed | On highs | Narrow-shallow + deep | Continuous | V-shaped |
| 239 | Almost leather -dry clay | Covered | Connected | On highs | Variable | Continuous | Flat bottom V-shaped |
| 240 | Leather- dry clay | Covered | Connected | Covered | Wide-deep + shallow | Continuous | Flat bottom V-shaped |
| 614 | Sand on limestone | Loose | Separated | On highs | Variable | Continuous | V-shaped |
| 219 | Limestone | Covered | Connected | Covered | Narrow-shallow | Continuous | Flat bottom U-shaped |
| 218 | Basalt | Covered | Connected | Covered | Variable | Continuous | V-shaped |
| 246 | Flint | Concentrated | Closed | On highs | Variable | Continuous | V-shaped |

**III.3.c.** **Pits**

Pits are lower areas that appear on the natural surface of the shell (S6 Table). Considering that friction is a process of material attrition, during sufficiently intense attrition high surfaces were reduced first, sometimes levelling down to the lowest point of the pit, until the pits were no longer evident and the surface became flat. Pits were also created during the frictional process by detachment of larger particles, however, this does not seem to be the case for the shells in this experiment. The case of the hard flint, where large, irregularly shaped pits were formed, might possibly be an exception (S6 Fig: d1-2). The abrasion of the shells against soft materials formed pits with rounded edges, while against harder materials, it caused the collapse of the edges. Such collapse resulted in an irregular, jagged edge, as did abrasion with limestone (S6 Fig: b1-2) or their truncation, which resulted in sharp edges, as in the case of basalt and flint (S6 Fig: c1-2, d1-2). The harder materials erased most of the smaller pits (possibly larger pits could have been leveled through abrasion, had the experiment lasted longer than 15 minutes).

**S6 Table:** Characteristics of the pits observed on the shells used in the controlled experiment.

|  | **Material in contact** | **Size** | **Shape** | **Distribution** | **Shape of margins** |
| --- | --- | --- | --- | --- | --- |
| 220 | Flax fibers | No alteration | Irregular | No alteration | Rounded |
| 545 | Loose sand | No alteration | No alteration | No alteration | No alteration |
| 577 | Loose sand with water | Variable | Irregular | Dense | Rounded |
| 221 | Oiled leather | No alteration | No alteration | No alteration | No alteration |
| 222 | Leather | Large + variable | Irregular | Loose scattering | Rounded |
| 217 | Fresh papyrus | Large + variable | Irregular | Dense | Rounded |
| 223 | Dry wood log | Variable but large | Irregular | Loose scattering | Rounded |
| 239 | Almost leather-dry clay | Large | Irregular | Loose scattering | Rounded |
| 240 | Leather-dry clay | Large | Irregular | Loose scattering | Rounded |
| 614 | Sand on limestone | No pits | No pits | No pits | No pits |
| 219 | Limestone | Large | Round | Loose scattering | Irregular |
| 218 | Basalt | Large | Round | Loose scattering | Sharp |
| 246 | Flint | Large | Irregular | Loose scattering | Sharp |

**III.3.d. Polish**

The characteristics of the polish reflect several attributes that result from the properties of the material in contact (S7 Table). Polish may be evident to the naked eye, as in the case of papyrus sedge but its characteristics must be determined through high magnification. SEM observation can provide limited information on the properties of the microtopography when compared to reflected light microscopes, but the characteristics of reflectivity and opacity cannot be seen. Polish caused by a variety of materials was documented following the experiment.

**S7 Table**: Characteristics of the polish on the shells used in the controlled experiment

|  | **Material in contact** | **Microtopography** | **Distribution** | **Linkage** | **Reflectivity** | **Incidence** | **Texture** | **Opacity** |
| --- | --- | --- | --- | --- | --- | --- | --- | --- |
| 220 | Flax thread | Sinuous | Spread | Linked | Dull | On highs + on lows | Rough | Opaque + transparent |
| 545 | Loose sand | Irregular | Capillar | None | Bright | On highs | X | X |
| 577 | Loose sand with water | Sinuous | Reticular | Linked | Bright | On highs | Rough | Opaque |
| 221 | Oiled leather | Irregular | Spread | Spread | Dull | Spread | Rough | Transparent |
| 222 | Leather | Sinuous | Spread + patches | Linked | Bright | On highs | Smooth | Opaque |
| 217 | Fresh papyrus | Sinuous | Reticular | Linked | Bright | On highs | Smooth | Opaque |
| 223 | Dry wood | Sinuous-flat | Reticular + domes | Linked | Dull | On highs | Rough | Opaque |
| 239 | Almost leather- dry clay | Sinuous-flat | Spread + patches | Linked | Bright | On highs | Rough | Opaque |
| 240 | Leather -dry clay | Sinuous-flat | Spread | Spread | Dull | Spread | Rough | Opaque |
| 614 | Sand on limestone | Flat | Linear | None | Bright | Not applicable | Rough | Opaque |
| 219 | Limestone | Irregular | Spread | Spread | Dull | Covered | Rough | Transparent |
| 218 | Basalt | Flat | Linear | None | Bright | On high  + on low | Rough | Opaque |
| 246 | Flint | Flat | Linear + patches | Linked | Bright | On highs | Smooth | Opaque |

**Flax thread (S4 Fig: a1-2):** No changes were visible to the naked eye. The abrasion of the flax caused no substantial change in the topography, except for slight rounding of protruding surfaces, defined as sinuous. At a magnification of 100x, a dull, rough polish, appearing transparent, was spread over the entire surface. On the protruding surfaces it was slightly more developed and appears opaque.

**Loose sand (S4 Fig: b1-2):** No polish was evident to the naked eye. At magnification of 100x delicate polish was observed distributed in a capillary pattern, slightly developed on elevated surfaces. Polish developed to a very low degree, therefore texture and opacity could not be determined even at higher magnification of 200x.

**Loose sand and water (S4 Fig: c1-2):** Polish was evident to the naked eye. At a magnification of 100x the polish was bright, domed and linked by reticulation, with a rough texture, appearing opaque. This experiment clearly shows the enhancing abrasiveness of water, forming a highly developed polish in comparison to the experiment of abrading the shell on loose sand alone.

**Oil-soaked leather (S7 Fig: a):** Almost no polish was visible to the naked eye on the shell. At a magnification of 100x, a delicate transparent polish on protruding surfaces and low areas was observed. The polish was rough in texture and dull in reflection.

**Dry leather (S4 Fig: d1-2):** No obvious change was seen by the naked eye. At a 100x magnification, smooth, bright, extremely domed polish that developed on protruding surfaces was observed. This polish is significantly different from the polish produced by the contact with oiled leather. Notably, during the experiment the shell heated and became warm to the touch, a process that we did not encounter with any of the other materials.

**Papyrus sedge (S5 Fig: a1-2):** Polish was visible to the naked eye. At a magnification of 100x, the area with polish was sinuous where bright smooth polish had developed on the rounded protruding surfaces, with many pits still visible.

**Almost leather-dry clay (S5 Fig: b1-2):** Polish was clearly visible to the naked eye. At a magnification of 100x, bright, rough opaque polish could be seen, with sinuous topography that developed mostly on the elevated surfaces.

**Leather-dry clay (S5 Fig: c1-2):** In this experiment, the outcome was polish that was clearly visible to the naked eye. At a magnification of 100x the polish appeared dull, rough and opaque, spreading throughout the surface, which was flat yet rounded at the edges of the polished surfaces. As in the case of the loose sand experiment, it was evident that water served as a lubricant that enhanced abrasion. The humid, almost leather-dry clay produced polish that developed to a higher degree and exhibited higher reflectivity than the leather-dry clay.

**Dry wood (S5 Fig: d1-2)**: Polish was clearly visible to the naked eye. At a magnification of 100x, a dull, rough polish was evident, distributed as wide flat polished surfaces that appeared domed because they were rounded on the edges. These domes were linked, thus creating a pattern of reticulation combined with domes.

**Loose sand on limestone (S6 Fig: a1-2):** In this experiment the surface was observed in intervals of 60 motions in order to follow changes that may have occurred during the progression of the frictional process. Following the experiment, no polish was evident to the naked eye, but a clear change in the properties of the natural surface was observed, mainly exhibiting a heavily grooved surface. Throughout the experiment the wear pattern did not change. The only change was the reduction in thickness of the worked surface, suggesting that at some point the surface would collapse and a hole would form. The polish formed is linear, spreading in isolated locations closely associated with the linear features. It is flat, bright and opaque.

**Limestone (S6 Fig: b1-2):** The experiment resulted in the abraded surface being covered with residue (shell and limestone powder). The shell was first rinsed in cold water, then soaked in water for an hour, and finally placed in an ultrasonic tank for five minutes to remove the residue. At a magnification of 100x, polish was evident, defined as flat, rough, matt and transparent in appearance. The natural surface was entirely altered.

**Basalt stone (S6 Fig: c1-2):** Following the experiment, a strong sheen on the shell was visible to the naked eye. At a magnification of 100x, we observed that the natural surface was drastically altered, with only few tiny pits remaining as evidence for low surfaces of the natural surface. The polish was evident between the striations that densely cover the surface, appearing as linear, flat, bright and opaque polish. There were few tiny rounded pits as a result of the bi-directional motion.

**Flint (S6 Fig: d1-2):** After the experiment was performed, polish was clearly visible to the naked eye. At a magnification of 100x, the polish was extremely flat, distributed in linear patches, very bright and opaque. This pattern exhibits a typical example that can be attributed to a very hard material.

To sum up, the controlled experiment showed that shells are easily affected by abrading substrates and can bear particular deformations that reflect tribological mechanisms (mechanisms of wear and friction), especially polish, which provided a large number of attributes. The interrelations between these and other attributes detailed above are crucial for determining the material in contact with the shells as exemplified by our experiment. The variations are numerous, and accord with the different characteristics of the materials in contact with the shells. Thus a careful study of the use-wear will result in a detailed dataset for interpretation of traces observed on archaeological artifacts. It is evident that polish characteristics result from several aspects associated with the process of abrasion, affected by hardness, elasticity, humidity, grain size and compactness. Some of the characteristics may be similar, for example, a sinuous pattern of microtopography typical of soft materials, but each case also exhibits uniqueness, for example, the oiled leather, where an irregular pattern was observed because alteration was very slow. It is therefore evident that wear patterns are correlated to specific mechanisms closely associated with the particular materials worked thus enabling a profound understanding of the wear mechanisms. Notably, SEM observations reduce accuracy because some of the properties (reflectivity, opacity) are less distinct and cannot be seen, thus reducing the chance of distinguishing the material in contact. This suggests that SEM is not required for future similar observations on shell manipulation.

**III.4. Results of the ornamental binding experiment**

**III.4.a. Traces from contact with a string**

Following the experiment of a thread suspended through the hole of a shell, only minor changes were observed in the morphology of the margin of the hole. Traces were detected only with high magnification, from 100x upward. Except for the experiment with the shell hung loosely, all shells exhibit traces of contact with string (S8 Fig). It is assumed that in the case of the loosely hung thread there was insufficient contact due to the light weight of the shell, therefore traces did not develop. On the other hand, knotted shells and shells tied together were subjected to a more intense contact because the string was tight against the shells' surface, which enhanced abrasion, thus causing traces to be formed more rapidly.

The binding traces appear as delicate striations resulting from contact with the soft and flexible flax thread. Traces were located on the circumference of the hole (S9 Fig: a-b), indicating the point where the string is threaded, similar to the position observed on the Qafzeh shells. Striations were oriented perpendicular to the hole, parallel to the long axis of the valve, also indicating the direction of the thread.

**III.4.b. Shell-to-shell contact**

Traces of shell-to-shell contact resulted in isolated patches of polish. It was observed that by following the location of the polish patches, it might be possible to reconstruct the mode by which the shells were tied together.

The first pattern corresponds to an exterior-to-interior valve arrangement that includes polish patches along the interior of the valve margin and polish patches on the exterior face of the shell near the margin (S9 Fig: c-d, f).

The second pattern corresponds to an interior-to-interior arrangement and resulted in patches of polish along the margin, seen on the interior face of the valve. Although this pattern might be confused with the former, it is different in that the polish patches are on the edge of the margin, not so much in the inner part of the interior face, as would be expected in the former pattern (S9 Fig: e).

**References**

61. Jelinek AJ, Farrand WR, Hass G, Horowitz A, Goldberg P. New excavations at the Tabun cave, Mount Carmel, Israel: A preliminary report. Paléorient 1973;1: 151-183.

62. Weinstein‑Evron M, Tsatskin A. The Jamal cave is not empty: Recent excavations in the Mount Carmel Caves, Israel. Paléorient 1994;20(2): 119-128.

63. Weinstein-Evron M, Bar-Oz G, Zaidner Y, Tsatskin A, Druck D, Porat N, et al. Introducing Misliya Cave, Mount Carmel, Israel: A new continuous Lower/Middle Paleolithic sequence in the Levant. Eurasian Prehistory 2003;1(1): 31-55.

64. Zaidner Y, Weinstein-Evron M. Making a point: The Early Mousterian toolkit at Misliya Cave, Israel. Before Farming 2014;2014: 1-23.

65. Meignen L, Bar-Yosef O. Middle Palaeolithic Lithic Variability in Kebara Cave, Mount Carmel, Israel. In: Akazawa T, editor. The Evolution and Dispersal of Modern Humans in Asia. Tokyo: University of Tokyo; 1992. pp 129-148.

66. Bar-Yosef O. The chronology of the Middle Palaeolithic of the Levant. In: Akazawa T, Aoki K, Bar-Yosef O. editors. Neandertals and Modern Humans in Western Asia. New York: Plenum press; 1998. pp 39-56.

67. Mercier N, Valladas H. Reassessment of TL age estimates of burnt flints from the Palaeolithic site of Tabun cave, Israel. Journal of Human Evolution 2003;45: 401-409.

68. Mercier N, Valladas H, Frojet L, Joron J-L, Ryess J-L, Weiner S, et al. Hayonim cave: A TL-based chronology for this Leva tine Mousterian sequence. Journal of Archaeological Science 2007;34(7): 1064-1077.

69. Meignen L, Bar-Yosef O. The Acheulo-Yabrudian and Early Middle Paleolithic at Hayonim cave, (western Galilee, Israel): Continuity or break?. Journal of Human Evolution. 2020;139: Forthcoming. <https://doi.org/10.1016/j.jhevol.2019.102733>

70. Angelbeck G. Stone tools and shellfish: Ancient shellfish harvesting on the former Transkei coast. The Digging Stick, Newsletter of the South African Archaeological Society 2011;28(2): 11-13.

71. Orton J, Jerardino A, Halkett D. On limpets, their height, and how to get them on the plate: A response to Angelbeck. The Digging Stick, Newsletter of the South African Archaeological Society 2012;29(1): 13-14.

72. Reese D. Middle Palaeolithic Shells from Ras el-Kelb. In: Copeland L, Moloney N, editors. The Mousterian site of Ras el-Kelb, Lebanon. Oxford: BAR International Series 706;1998. pp 67.

73. Colonese AC, Mannino MA, Bar-Yosef Mayer DE, Fa D, Finlayson JC, Lubell D, et al. Marine mollusc exploitation in Mediterranean prehistory: An overview. Quaternary International 2011;239: 83-103.

74. Villa P, Soriano S, Pollarolo L, Smriglio C, Gaeta M, D’Orazio M, et al. Neandertals on the beach: Use of marine resources at Grotta dei Moscerini (Latium, Italy). PLoS ONE 2020;15(1): e0226690.

75. Bailey GN, Flemming NC, King GCP, Lambeck K, Momber G, Moran LJ, et al. Coastlines, submerged landscapes, and human evolution: The Red Sea Basin and the Farasan islands. Journal of Island and Coastal Archaeology 2007;2: 127-160.

76. Heller J, Sivan N, Ben-Ami F. Systematics of *Melanopsis* from the coastal plain of Israel (Gastropoda: Cerithioidea). Journal of Conchology 2002;37(6): 589-606.

77. Hunt CO, Hill EA. Caves and molluscs. In: Allen MJ, editor. Molluscs in Archaeology. Oxford: Oxbow; 2017. pp 100-110.

78. Yeshurun R, Bar-Oz G, Weinstein-Evron M. Modern hunting behavior in the Early Middle Paleolithic: Faunal remains from Misliya Cave, Mount Carmel, Israel. Journal of Human Evolution 2007;53: 656-677.

79. Texier PJ, Porraz G, Parkington J, Rigaud JP, Poggenpoel C, Miller C, et al. A Howiesons Poort tradition of engraving ostrich eggshell containers dated to 60,000 years ago at Diepkloof rock shelter, south Africa. Proceedings of the National Academy of Science 2010;107(14): 6180-6185.

80. Groman-Yaroslavski I, Zaidner Y, Weinstein-Evron M. Mousterian Abu Sif points: Foraging tools of the Early Middle Paleolithic site of Misliya cave, Mount Carmel, Israel. Journal of Archaeological Science: Reports 2016;7: 312-323.

81. Leontarakis PK, Xatzianastasiou LI, Theodorou JA. Biological aspects of the lagoon cockle, *Cerastoderma glaucum* (Poiret 1879), in a coastal lagoon in Keramoti, Greece in the northeastern Mediterranean. Journal of Shellfish Research 2008;27(5): 1171-1175.

82. Sivan D, Greenbaum N, Cohen-Seffer R, Sisma-Ventura G, Almogi-Labin A. The origin and disappearance of the late Pleistocene–early Holocene short-lived coastal wetlands along the Carmel coast, Israel. Quaternary Research 2011;76: 83-92.

83. Fishelson L. Toxicological aspects associated with the ecology of *Donax trunculus* (bivalvia, mollusca) in a polluted environment. The Science of the Total Environment 1999;226(2-3): 121-131.

**List of figures: SUPPLEMENTARY**

S1 Fig: The controlled experiment showing most of the materials used to abrade the shells: a: fresh papyrus, b: flax thread, c: loose sand, d: dry wood log, e: leather, f: almost leather-dry clay, g: leather-dry clay, h: sand on limestone, i: basalt, j: limestone, k: flint. (Credit: Use wear Laboratory, Zinman Institute of archaeology, Iris Groman-Yaroslavski).

S2 Fig: The ornamental binding experiment showing the mode by which shells were hung: a: a shell hanging loosely on a string, b: a shell knotted to a string, c: shells tied in ventral-to-dorsal configuration, d: shells tied in a ventral-to-ventral configuration, e: the setting of the experiment with the shell hanging in front of a fan. (Credit: Use wear Laboratory, Zinman Institute of archaeology, Iris Groman-Yaroslavski).

S3 Fig: Micrographs showing the natural wear pattern of shells collected on the beach: a: polish developed on elevated surfaces of the rough topography, b: isolated grooves (original magnification 100x). (Credit: Use wear Laboratory, Zinman Institute of archaeology, Iris Groman-Yaroslavski).

S4 Fig: Use-wear produced by the contact with soft materials observed on shells from the controlled experiment comparing micrographs by reflected light microscope on left row to SEM on right row: a:1-2: flax thread, b:1-2: loose sand, c:1-2: loose sand and water, d:1-2: leather (original magnifications 100x). (Credit: Use wear Laboratory, Zinman Institute of archaeology, Iris Groman-Yaroslavski).

S5 Fig: Use-wear produced by the contact with materials of medium hardness observed on shells from the controlled experiment comparing micrographs by reflected light microscope on left row to SEM on right row: a:1-2: fresh papyrus, b:1-2: almost leather-dry clay c:1-2: leather dry clay, d:1-2: dry wood log (original magnifications 100x). (Credit: Use wear Laboratory, Zinman Institute of archaeology, Iris Groman-Yaroslavski).

S6 Fig: Use-wear produced by the contact with hard materials observed on shells from the controlled experiment comparing micrographs by reflected light microscope on left row to SEM on right row: a:1-2: sand on limestone, b:1-2: limestone, c:1-2: basalt, d:1-2: flint (original magnifications 100x). (Credit: Use wear Laboratory, Zinman Institute of archaeology, Iris Groman-Yaroslavski).

S7 Fig: Micrographs showing use-wear produced in the controlled experiment comparing traces produced by abrading oiled leather (a substance replicating hide with fats at a progressive stage of processing) (a) to dry leather (b). (Credit: Use wear Laboratory, Zinman Institute of archaeology, Iris Groman-Yaroslavski).

S8 Fig: Micrograph showing the striations produced by binding observed on the experimental shell (original magnification 100x). (Credit: Use wear Laboratory, Zinman Institute of archaeology, Iris Groman-Yaroslavski).

S9 Fig: *Glycymeris* shells with indication (black squares) showing the location of traces by binding (a-b) and the ideal distribution of patches of polish produced by contact of shell to shell in a dorsal-to-ventral arrangement (c-d), ventral-to-ventral arrangement (e) and dorsal-to-dorsal arrangement (f). (Credit: Use wear Laboratory, Zinman Institute of archaeology, Iris Groman-Yaroslavski).


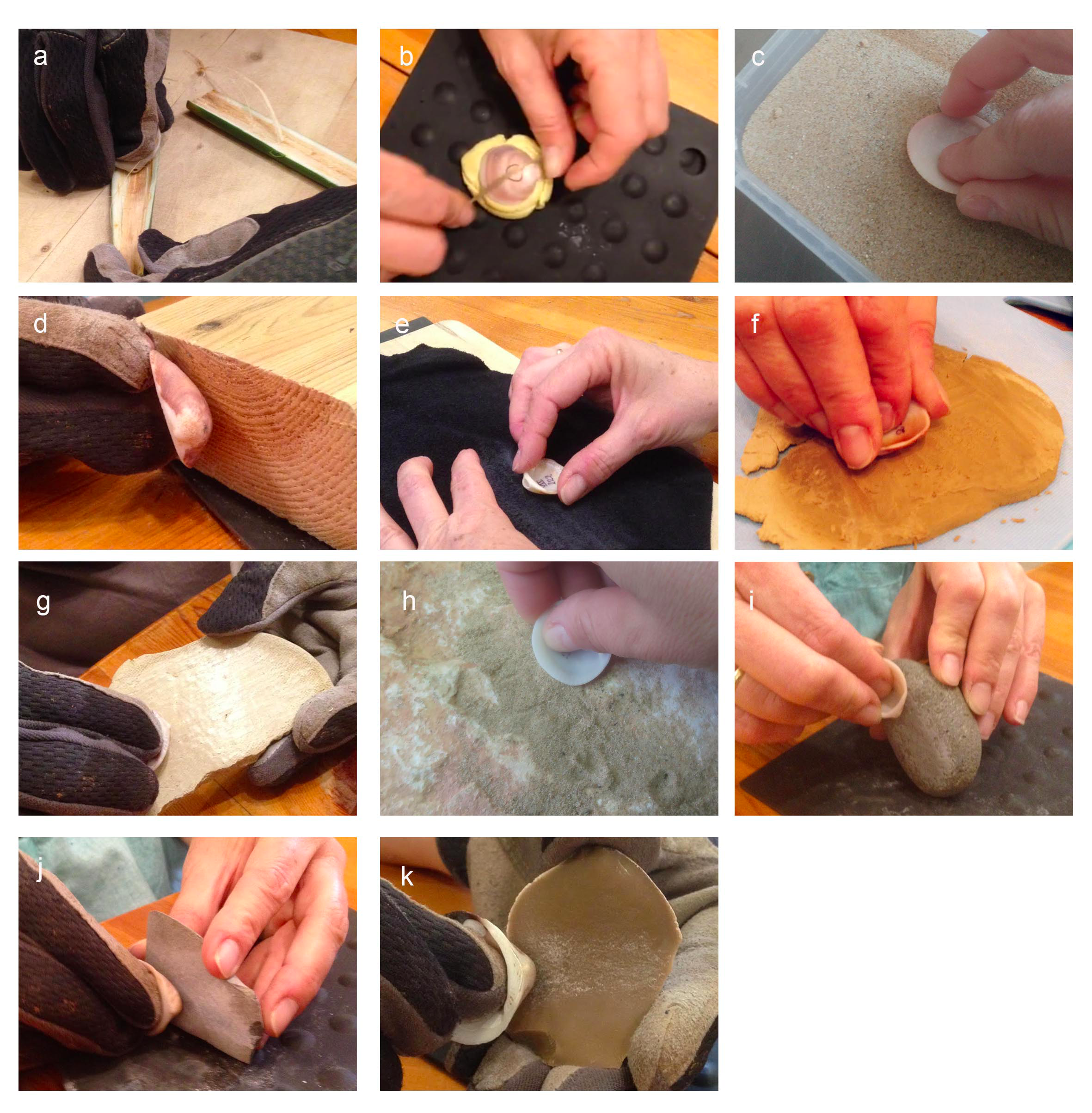


S1 Fig


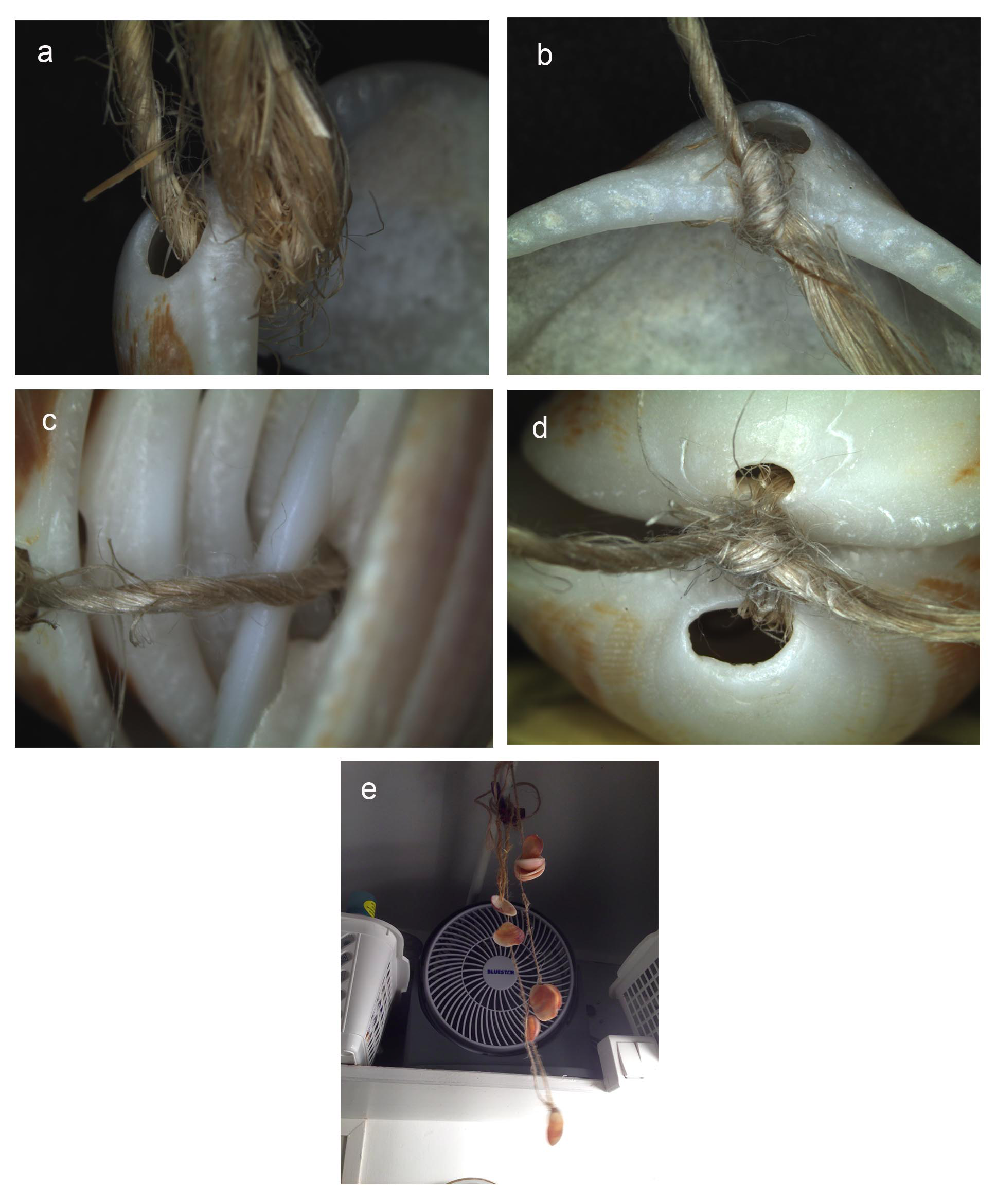


S2 Fig


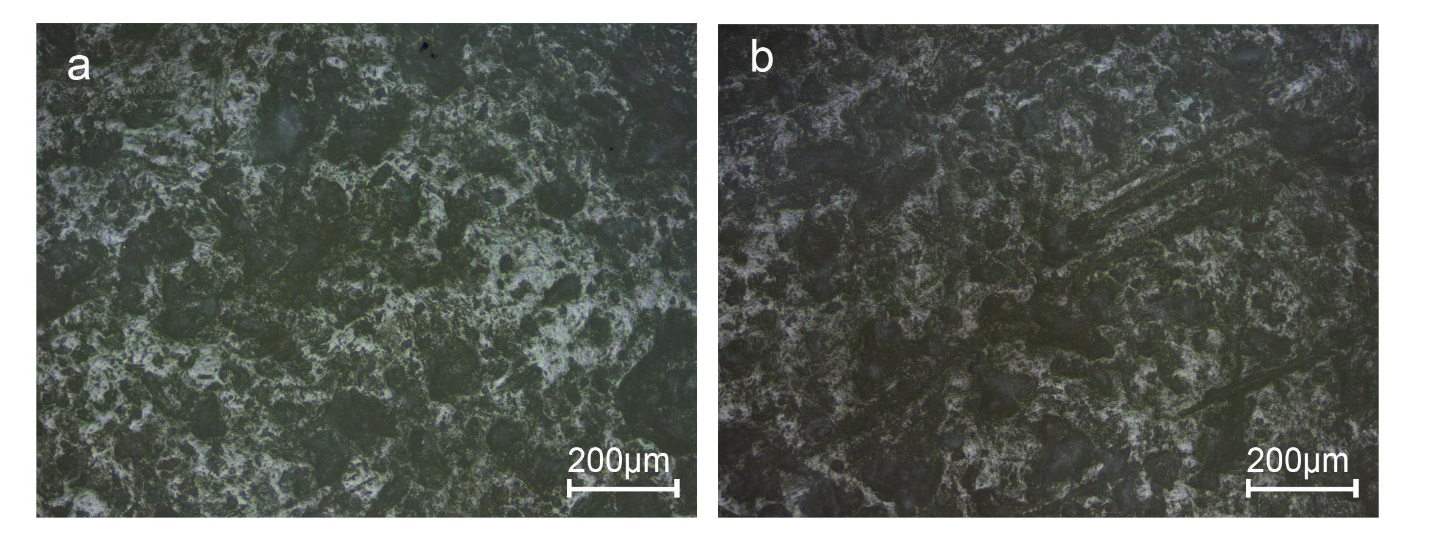


S3 Fig


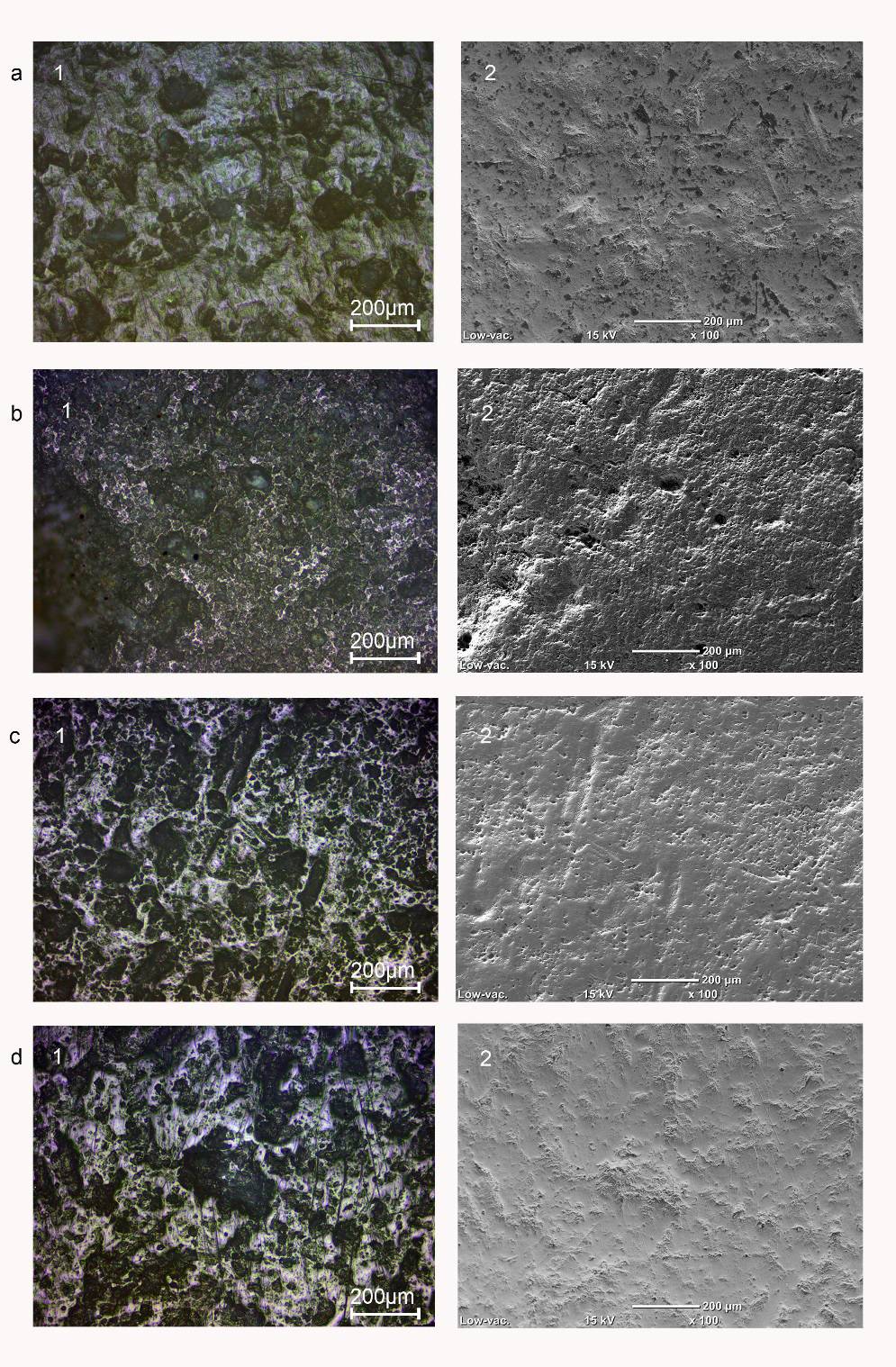


S4 Fig


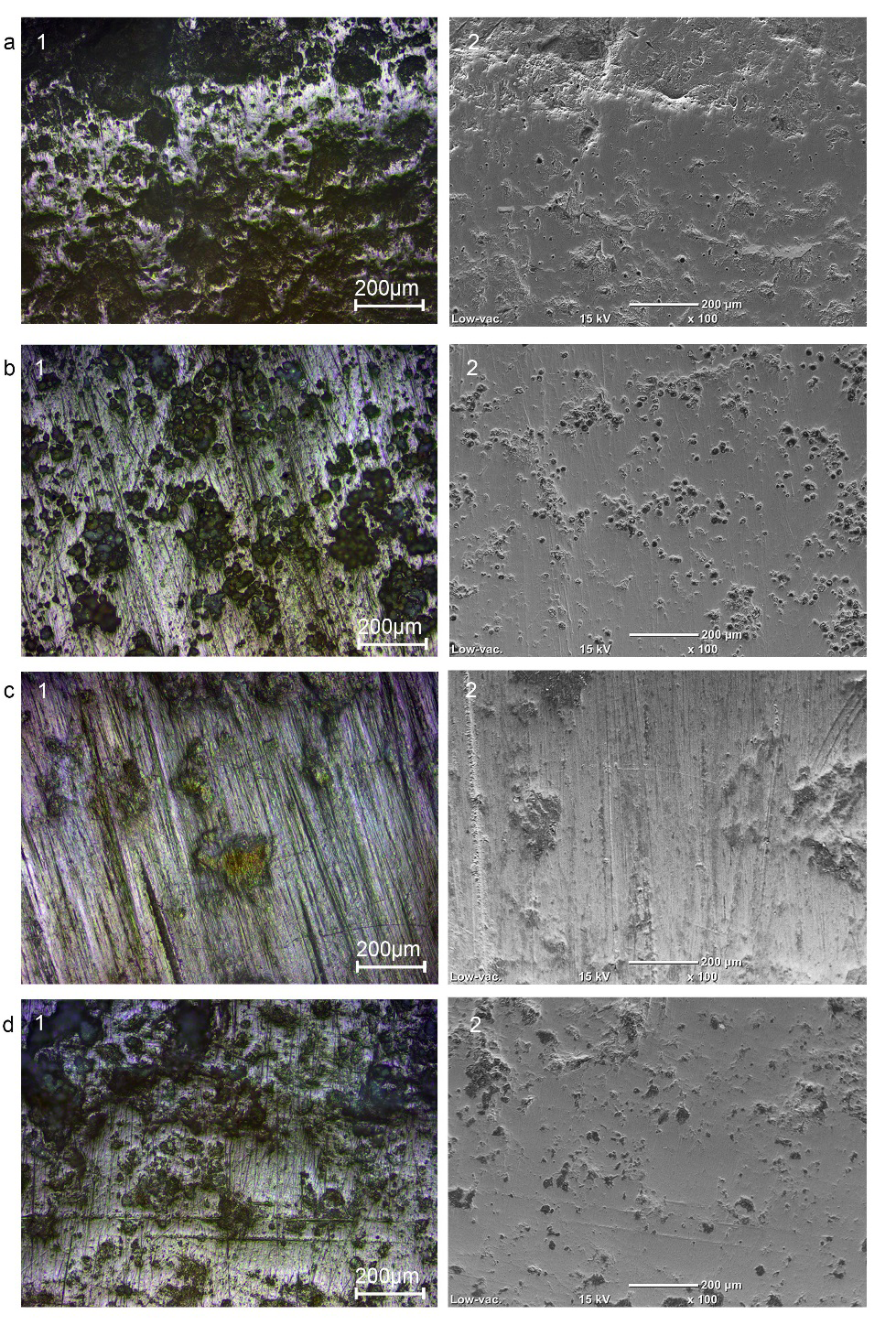


S5 Fig


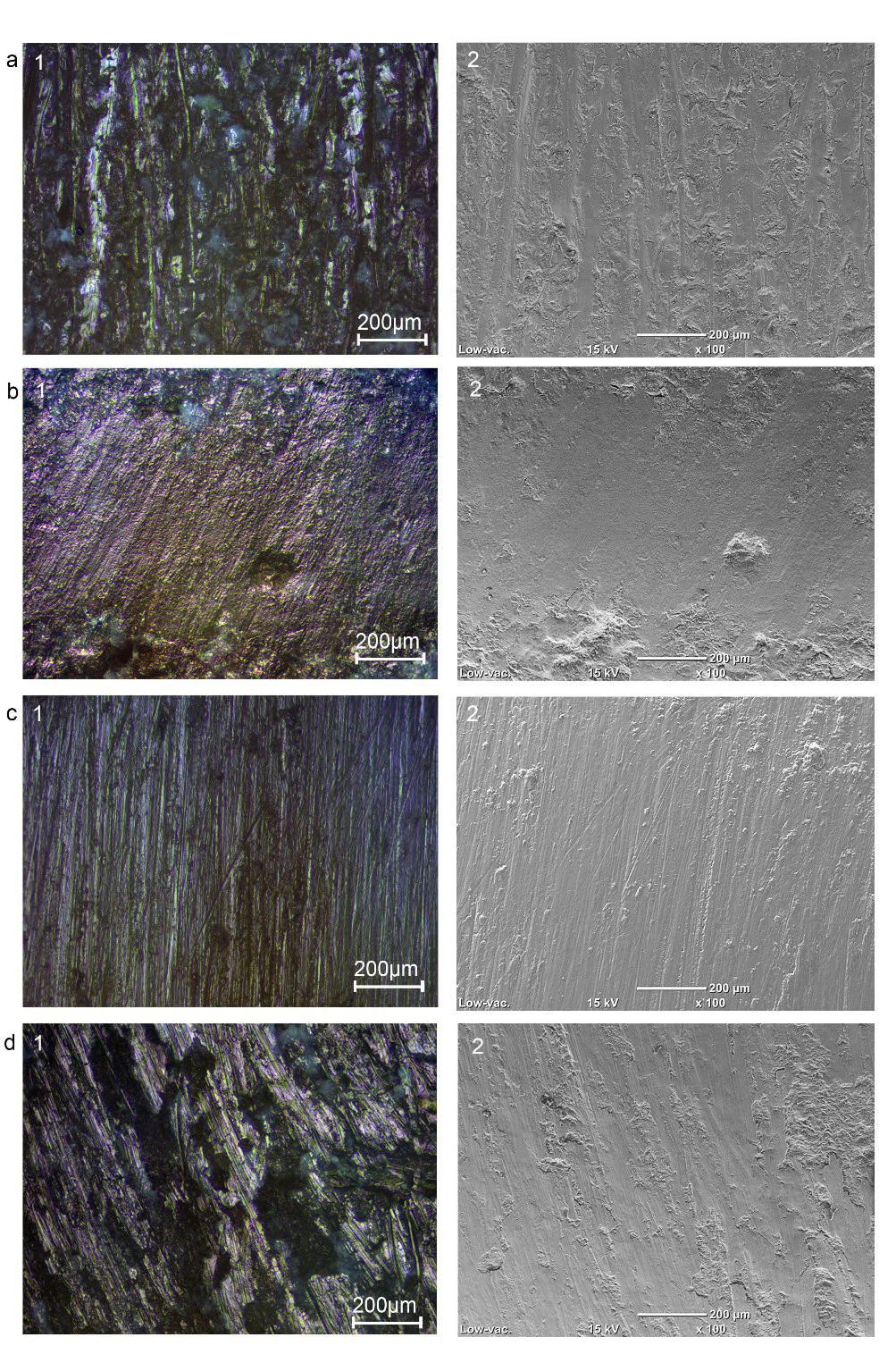


S6 Fig


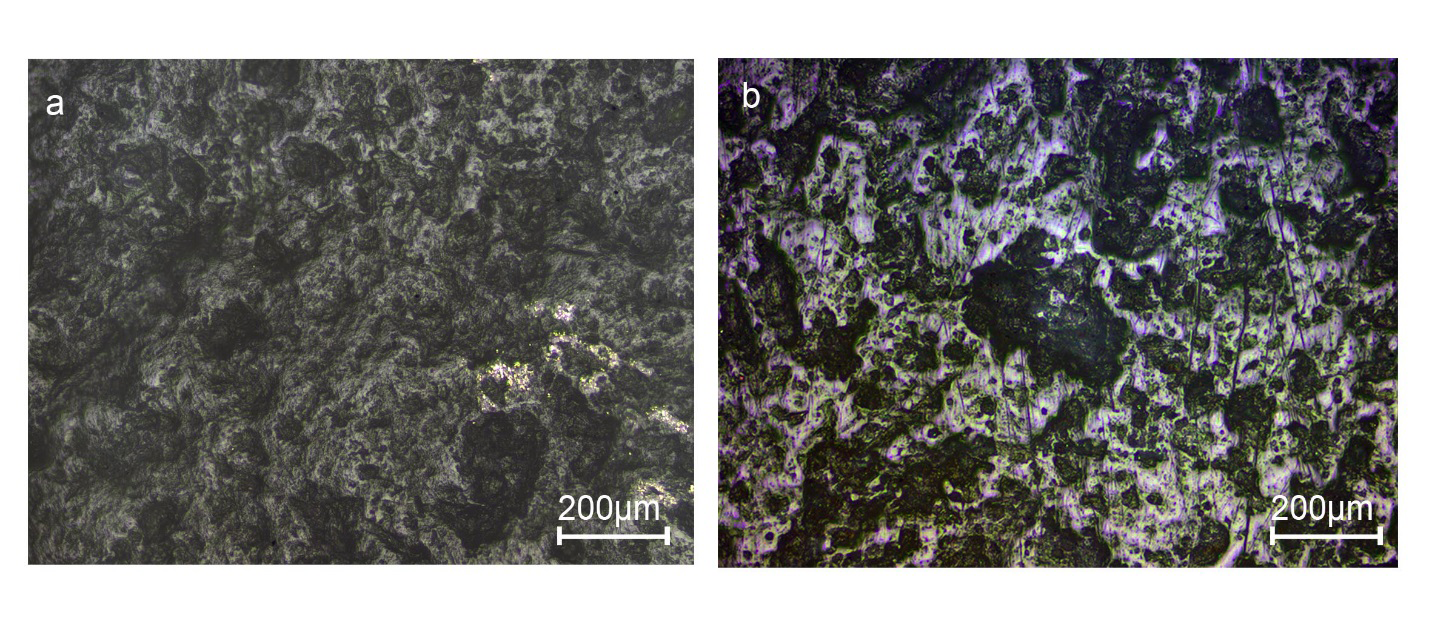


S7 Fig


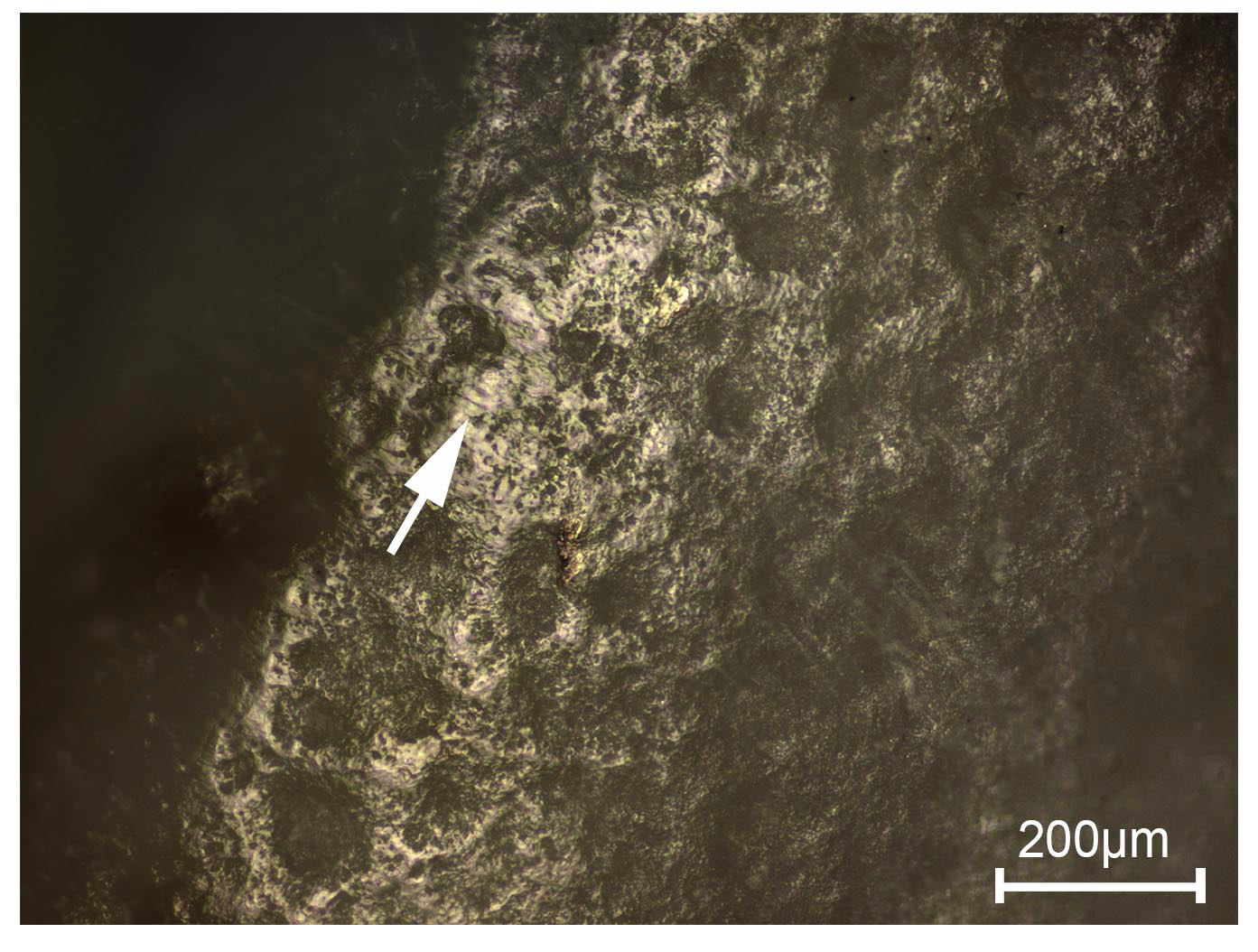


S8 Fig


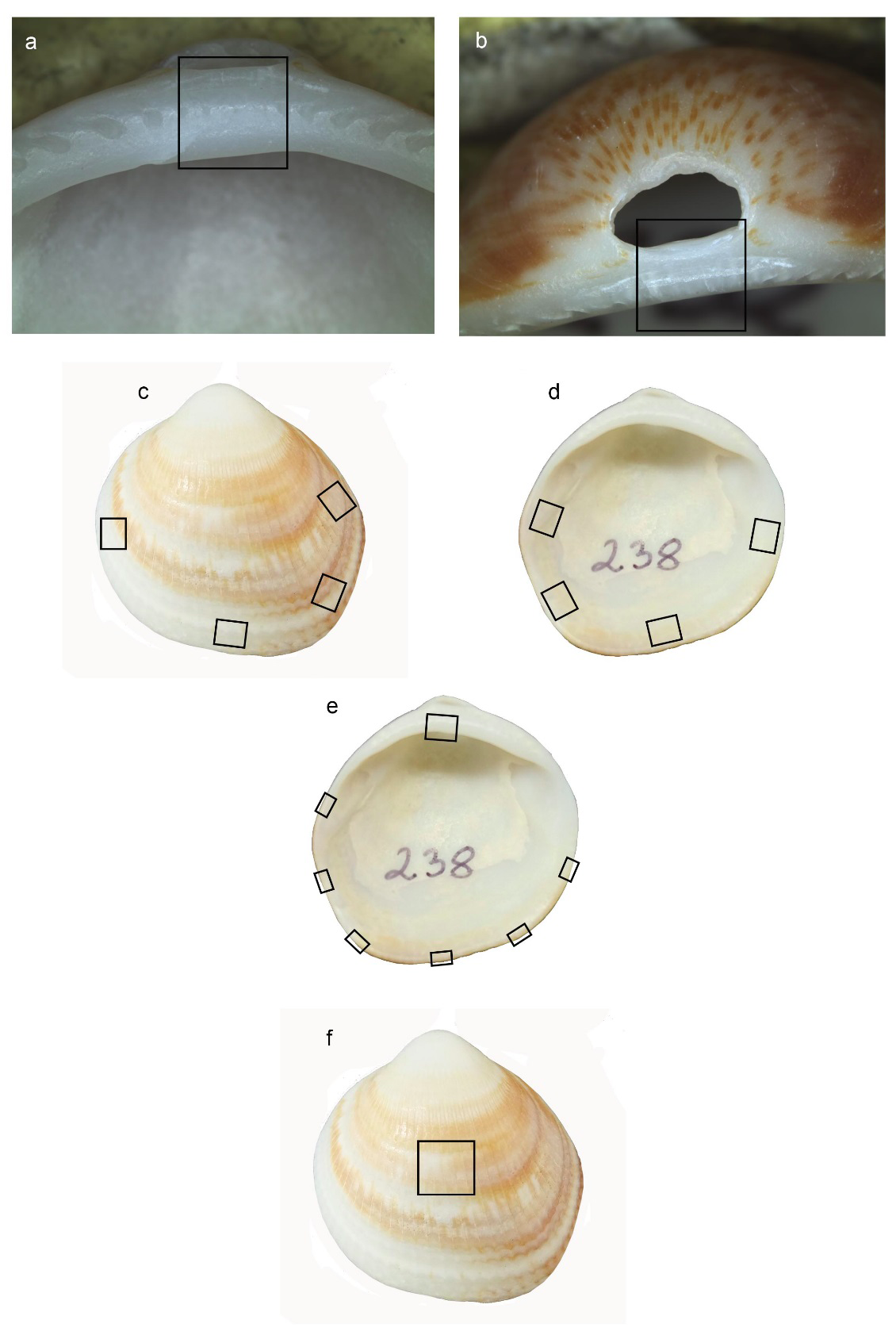


S9 Fig
